# Spectral graph modeling reveals global slowing of neurophysiological network transmission in Alzheimer’s disease

**DOI:** 10.21203/rs.3.rs-2579392/v1

**Published:** 2023-03-22

**Authors:** Parul Verma, Kamalini Ranasinghe, Janani Prasad, Chang Cai, Xihe Xie, Hannah Lerner, Danielle Mizuiri, Bruce Miller, Katherine Rankin, Keith Vossel, Steven W. Cheung, Srikantan Nagarajan, Ashish Raj

**Affiliations:** 1 Department of Radiology and Biomedical Imaging, University of California San Francisco, San Francisco, CA, USA; 2 Memory and Aging Center, Department of Neurology, University of California San Francisco, San Francisco, CA, USA; 3 Amador Valley High School, Pleasanton, CA, USA; 4 Mary S. Easton Center for Alzheimer’s Research and Care, Department of Neurology, David Geffen School of Medicine, University of California Los Angeles, Los Angeles, CA, USA; 5 Department of Otolaryngology-Head and Neck Surgery, University of California San Francisco, San Francisco, CA, USA; 6 Surgical Services, Veterans Affairs, San Francisco, USA

**Keywords:** brain activity, Alzheimer’s disease, magnetoencephalography, spectral graph theory, cognitive decline

## Abstract

Alzheimer’s disease (AD) is the most common form of dementia, progressively impairing memory and cognition. While neuroimaging studies have revealed functional abnormalities in AD, how these relate to aberrant neuronal circuit mechanisms remains unclear. We employed a spectral graph-theory model (SGM) to identify abnormal biophysical markers of neuronal activity in AD. SGM is an analytic model that describes how long-range fiber projections in the brain mediate excitatory and inhibitory activity of local neuronal subpopulations. We estimated SGM parameters that captured the regional power spectra obtained from magnetoencephalography imaging of a well-characterized population of patients with AD and controls. The long-range excitatory time constant was the most important feature for the accurate classification of AD and controls and was associated with global cognitive deficits in AD. These results indicate that a global impairment in the long-range excitatory neurons might be a sufficient factor underlying spatiotemporal alterations of neuronal activity in AD.

## 1 Introduction

Alzheimer’s disease (AD) is the most common form of dementia, progressively impairing the cognition and behavior of the affected individual. It has been proposed that the effect of AD neurodegeneration on cortical neuronal networks is partially reflected by the abnormal mechanisms of cortical neural synchronization and coupling [1]. Neural synchronization refers to the simultaneous activity of neuronal groups in the brain. Repetitive spiking activities of neural populations form an oscillatory behavior at frequencies ranging from slow delta waves to fast gamma waves. We may distinguish between two types of synchrony: *local synchrony*, typically measured via the power spectral density (PSD) of the electrophysiological signal at the regional level; and *long-range synchrony*, deduced as the pair-wise coherence between signals originating at different locations - also referred to as *functional connectivity* or FC. Neurodegeneration is thought to disrupt both local and long-range synchronization [2, 1, 3]. These deficits are present long before the onset of clinical symptoms, worsen as the symptoms occur and progress, and may play a role in disease manifestation [4]. It is therefore important to understand how the pathology-induced neurodegeneration in AD leads to disruption in large-scale synchrony within and between brain regions, and how they then lead to cognitive impairment.

Functional neuroimaging studies are indispensable for understanding the role of aberrant synchrony in AD, and of its functional consequences. Resting-state functional MRI (fMRI), which measures the slow fluctuations of blood oxygenation signal in the brain as a proxy for neural activity [5], readily gives the low-frequency FC. Leveraging graph-theoretic analyses, many studies now routinely describe the alterations in FC during the course of AD pathophysiology [6]. Graph theoretic statistics of FC from resting-state fMRI however have shown inconsistent differences between AD patients and control samples [7, 8], perhaps due to fMRI’s limited ability to capture fast temporal scales of neuronal activity [9].

In contrast, electrophysiological techniques such as electro- or magneto-encephalography (E/MEG) can capture temporal activity scales with millisecond precision. FC features extracted from MEG activity were demonstrated as an early biomarker of AD burden [10–12] and showed that the graph metrics between AD and control samples vary across different frequency bands. Other studies have demonstrated aberrant FC signatures in AD [13–18]. Degradation of callosal fiber integrity was accompanied by loss of brain interhemispheric functional connectivity characterized by increased delta-band, and decreased alpha-band, path length using FC obtained from EEG recordings [19], suggesting that global FC measures can reflect fiber disconnectivity in AD. Significant differences were observed in various coherence-based measures of long-range synchrony in AD compared to control subjects [1]. There is also now substantial evidence that local synchrony is aberrant in AD. EEG analysis revealed that the relative PSD of AD patients is significantly increased in delta- and theta-, while reduced in alpha-band [2]. A recent MEG study conducted by our group suggested striking shifts in the power spectra of neural activity in AD patients compared to healthy subjects, the most prominent being a shift of power from the normally-dominant alpha band to the lower delta-theta bands [18]. We further showed that the shift in MEG spectra in AD is regionally-specific and accounted for by altered excitatory and inhibitory parameters which are well-predicted by local *Aβ* and tau concentration as measured by PET imaging - giving a direct connection to how pathology alters the excitatory-inhibitory balance in AD [18].

These data have naturally led to a search for underlying neural mechanisms whose impairment in AD might help explain these electrophysiological alterations in AD. A beginning in this direction was reported in our recent study [18], where each regional PSD was fitted by a localized and linearized neural mass model. While such local models can powerfully reveal local alterations in neural systems, e.g. excitatory/inhibitory imbalances, they did not consider the anatomic network structure of the brain. Indeed, the anatomic network enables short- and long-range communication between cortical neurons, and through regenerative feedback, can support spontaneous functional organization of synchronized neuronal firing at different timescales and generates oscillations of different frequencies [20]. Further, a local disruption of neural systems is not the only possible factor at play in AD. It is well known that AD is accompanied by global (i.e. brain-wide) changes in electrophysiological processes, e.g. slower axonal conductance speeds [21], less efficient signal transfer due to axonal swelling and demyelination [22], higher noise and less efficient synaptic signaling [23], across the brain. All these global effects have the potential to alter the spectral profile as well as the FC of neural activity measured on MEG.

Therefore there remains a pressing need to apply parsimonious models of brain activity that are applicable at the whole-network level while accommodating global, region-invariant parameters, and to understand whether such models can recreate AD-induced spectral shifts. In these efforts, it is frequently necessary to employ mathematical models that codify the effect of abnormal changes in biophysical mechanisms on neuronal activity. Computational investigations, frequently employing coupled neural mass models, are becoming popular in AD and dementia [24], yet their practical impact has been limited by their non-linearity, long simulation times, high dimensionality, and intractable parameter inference.

In order to address these challenges, in this paper we introduce a rigorous model-based assessment of the role of global, mechanistic alterations in AD electrophysiology, especially their coupling via the structural connectome. This approach, therefore, seeks to complement our prior study on uncoupled locally-varying neural masses [18]. The mathematical model we employed is the spectral graph theory-based model (SGM) to identify abnormal biophysical markers of neuronal activity in AD. SGM is a linearized and analytic (i.e. closed-form) model that describes the activity of excitatory and inhibitory neuronal populations in different brain regions, coupled and mediated by the long-range white matter structural wiring [25, 26]. SGM is parameterized by a small set of 7 global, regionally-invariant parameters, and relies on the connectome to achieve regional diversity. It was previously successful in capturing the resting state MEG spectral signatures across all brain regions of healthy subjects [25, 26]. Due to its parsimony, SGM yields easy and tractable model inference and hence is an ideal tool for inferring underlying biophysical correlates of AD-related MEG alterations. We use SGM to specifically test the hypothesis that the model’s parameters that control long-range synchrony via its coupling with the anatomical network will be impaired in AD and could provide a parsimonious account for cognitive decline.

We performed a thorough parameter inference of the SGM on individual subjects’ MEG recordings from a well-characterized clinical population of AD patients and a cohort of age-matched controls studied previously [17]. Detailed statistical analysis on the fitted parameters was conducted in order to assess group-level differences in global mechanisms of neural activity. We found that while AD and healthy groups showed many subtle differences in several global SGM parameters, only one stood out and was responsible for the majority of group-wise changes. Indeed, patients with AD have significantly elevated long-range excitatory neuronal time constant compared to controls. This time constant is also the most important feature for the accurate classification of AD from controls and is strongly associated with the global measure of cognition. Intriguingly, the SGM can be decomposed into a small set of eigenmodes of the network, in a striking resemblance with a similar eigendecomposition that we previously used to explain the spread of pathology on the brain [27]. Our results clearly implicate altered long-range excitatory time constant as a key hallmark of AD-related function. To our knowledge, this is the first report of a single global parameter change that can reliably produce spectral shifts from healthy to AD brains. Our study is therefore an intriguing counterpoint to prior studies highlighting the localized effects of AD on brain activity. Potentially, this fills a key missing link between AD pathology and downstream altered activity and cognitive deficits.

## 2 Results

SGM provides a closed-form solution of the steady-state frequency response of different brain regions. Here, we use a Desikan-Killiany parcellation scheme [28] to estimate the brain regions. The SGM is characterized by 7 parameters, which are either global or local but spatially-invariant. These parameters include the spatially-invariant local synchrony-related time constants *τ*_e_, *τ*_i_ and spatially-invariant but local neural gains *g*_*ei*_, *g*_*ii*_ at the mesoscopic scale for both excitatory and inhibitory neuronal subpopulations; as well as a global excitatory time constant *τ*_*G*_ at the macroscopic scale representing the long-range network connections, global coupling constant *α*, and speed of transmission of signals among regions *v*. Each region is assumed to consist of local excitatory and inhibitory neuronal subpopulations that interact with each other and regulate the macroscopic long-range excitatory neuronal populations. The macroscopic long-range populations are assumed to be connected to each other via the structural connectome. Here, we use a template structural connectome from the Human Connectome Project (HCP). Hence the model entails no features that may change from region to region, except of course features from the heterogeneously connected anatomical network.

**Figure 1:**
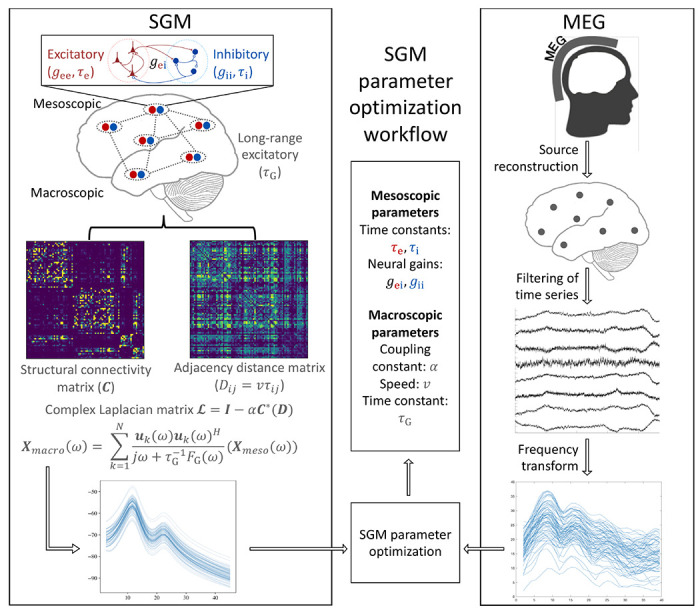
SGM models excitatory and inhibitory neuronal subpopulation signals that influence the long-range excitatory signals. The long-range signals are connected to each other via the structural connectome, and these signals transmit with a fixed conduction speed. SGM provides a closed-form solution in the frequency domain. This is compared to the frequency spectra obtained from MEG for inferring the SGM model parameters.

To infer the SGM parameters, we fit SGM output to the frequency spectra obtained from MEG for healthy controls and AD subjects. A workflow is shown in Fig. 1.

### 2.1 SGM reliably reproduces the patterns of spectral power density.

**Figure 2:**
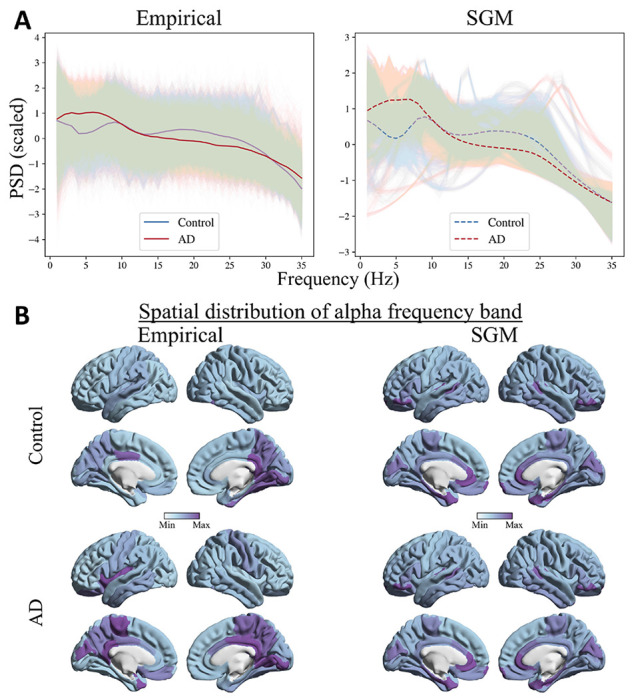
**A:** Comparison of empirical (left) and SGM (right) frequency spectra. Lighter lines correspond to frequency spectra for each brain region and subject. The darker lines correspond to the spectra averaged over all regions and subjects, separately for AD and controls. **B:** Spatial distribution of the empirical (left) and SGM (right) alpha frequency band, for subjects with mean spatial correlations in controls (top) and AD (bottom). The color scale of each spatial distribution was chosen based on their dynamic range.

The predicted spectra from SGM reliably captured the empirical MEG spectra from patients with AD and age-matched controls (Fig. 2A; The mean (std) spectral correlations were 0.72 (0.08) and 0.78 (0.09) for controls and AD, respectively). Compared to age-matched controls, patients with AD showed a reduced alpha peak and increased spectral power within the low-frequency delta-theta range (1-7 Hz), in their empirical spectral recording from MEG. This characteristic spectral change is clearly replicated in the predicted spectra derived from SGM. The spatial distribution of spectral power density of the alpha band, as expected, showed a postero-anterior distribution in both controls and patients. The spatial patterns of the predicted spectra from SGM reproduced this postero-anterior distribution with high fidelity (Fig. 2B; the mean (std) spatial correlations were 0.60 (0.09) and 0.66 (0.09) for controls and AD, respectively)

### 2.2 AD patients have altered network time constants and neural gains.

**Figure 3:**
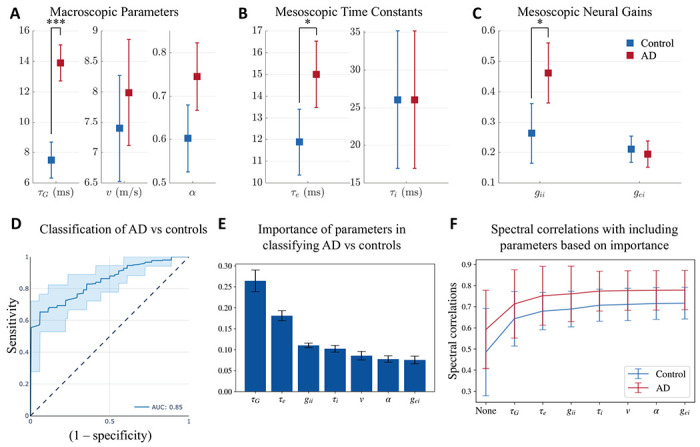
**A, B, C:** Statistical significance testing of difference in model parameters between AD and controls, with age as a covariate. Distribution of **A:** macroscopic parameters *τ*_G_ (long-range excitatory time constant), *v* (speed), and *α* (coupling constant); **B**: mesoscopic time constants *τ*_e_ (excitatory) and *τ*_i_ (inhibitory); and **C:** mesoscopic neural gains *g*_ii_ (inhibitory gain) and *g*_ei_ (gain of signals from the coupling between excitatory and inhibitory neurons). P-values are reported after correcting for multiple testing using a Bonferroni correction. *: *p* < 0.05, * * *: *p* < 0.001 **D, E:** Classification of AD vs controls with a random forest classifier with SGM parameters and age as features of the classifier. **D:** ROC curve for classification of AD versus controls. **E:** Feature importance plot of SGM parameters.**F:** Spectral correlations when optimizing for only certain model parameters while keeping the others fixed at the average of the optimized model parameters of both AD and controls. “None” implies that all the model parameters were fixed at the average. The second point on the x-axis with the label *τ*_G_ implies that only *τ*_G_ was allowed to be optimized while the other model parameters were fixed at the average values. The third point on the x-axis with the label *τ*_e_ implies that both *τ*_G_ and *τ*_e_ were allowed to be optimized while keeping the other model parameters fixed at the average values. All the subsequent points on the x-axis correspond to similarly including more model parameters in optimization, based on their importance in the classification of AD vs controls.

Next, we compared the network parameters derived from the SGM between patients with AD and age-matched controls. Recall that these parameters are either global or local but assumed spatially-invariant. Using a general linear model with age included as a covariate, we found that patients with AD have significantly elevated long-range excitatory time constant (*τ*_G_; controls mean = 7.50, confidence interval = (6.32, 8.68), AD mean = 13.90, confidence interval = (12.72, 15.09), Cohen’s D effect size = 1.16), mesoscopic excitatory time constant, (*τ*_e_; controls mean = 11.88, confidence interval = (10.36, 13.41), AD mean = 15.01, confidence interval = (13.48, 16.53), Cohen’s D effect size = 0.41) and mesoscopic inhibitory neural gain (*g*_ii_; controls mean = 0.26, confidence interval = (0.16, 0.36), AD mean = 0.46, confidence interval = (0.36, 0.56), Cohen’s D effect size = 0.42; Fig. 3A, B, and C). The highest effect size among the parameter comparisons was found in *τ*_G_ between AD and controls. Collectively these results indicate that while global network parameters are altered in AD, long-range excitatory connections may reflect such changes with greater sensitivity than other parameters.

### 2.3 Altered global network parameters can distinguish between AD and controls with high accuracy.

Next, we examined the sensitivity and specificity of altered global network parameters to distinguish between patients with AD and controls. To this end, we trained and tested a random forest classifier including the model parameters and age as the classifier features. The average AUC of the ROC curves from the testing folds is 0.85, with a standard deviation of 0.02 (Fig. 3D). The other classification metrics included: accuracy = 0.78, precision = 0.79, recall score = 0.75, and f1 score = 0.77, on average. We also obtained the feature importance score of the features used in training the model, shown in Fig. 3E. The time constant *τ*_*G*_ was the most important feature in classifying AD versus controls. Collectively, these results indicate that altered global network parameters are reliable indices to identify patients with AD from their age-matched counterparts and that long-range excitatory connections are the most sensitive indicators of AD-related global network deficits.

### 2.4 Minimal set of altered model parameters capture the empirical spectra.

In order to assess the importance of model parameters in capturing the empirical spectra, we evaluated the spectral and spatial correlations after optimizing for certain model parameters, based on their importance from Fig. 3E, while keeping the remaining model parameters as the average of all the optimized model parameters for AD and controls together. First, we evaluated the correlations when none of the model parameters are optimized for and are all the average of the optimal parameters obtained previously. Next, we optimized only for *τ*_G_ while keeping all the other model parameters fixed since *τ*_G_ was the most important parameter in the classification of AD vs controls. Subsequently, we optimized for both *τ*_G_ and *τ*_e_ while keeping the remaining model parameters fixed since *τ*_e_ was the second most important feature in classification. We repeated this procedure till we included all the model parameters for optimization. The spectral correlations from this evaluation are reported in Fig. 3F. As seen in the figure, we see a sharp increase when *τ*_G_ is allowed to vary while keeping the other model parameters fixed. Upon including the subsequent model parameters, we do not see a substantial increase in the spectral correlation. This result strengthens our prior observation on the importance of *τ*_G_ in differentiating AD from controls. Note that we did not see any substantial difference in the spatial correlations.

### 2.5 Altered long-range excitatory connections are correlated with global cognitive deficits in patients with AD.

**Figure 4:**
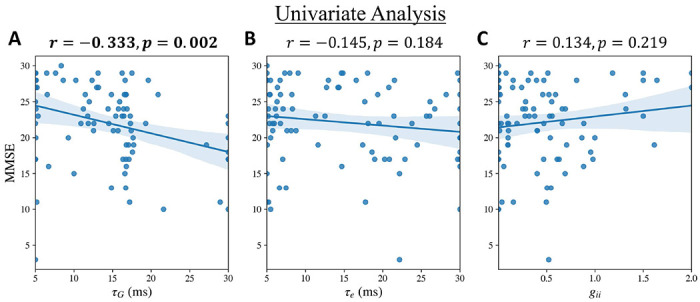
Univariate associations of **A**: *τ*_G_, **B**: *τ*_e_, and **C**: *g*_ii_ with MMSE in patients with AD.

To investigate the association between altered global network parameters and cognitive deficits in patients with AD, we examined the correlations between *τ*_G_, *τ*_e_, and *g*_ii_ with global cognitive decline measured by Mini Mental State Exam (MMSE), and overall disease severity measured by clinical dementia rating sum of boxes (CDR), in patients with AD. We first tested for univariate associations between the model parameters and MMSE and CDR separately, using linear regression. After adjusting for multiple testing (Bonferroni), *τ*_G_ showed significant negative associations with MMSE (Fig. 4A) where higher *τ*_G_ predicted greater cognitive deficits in MMSE. Next, we tested for the association between *τ*_G_ and MMSE including *τ*_e_, *g_ii_*, and age as covariates in a multivariate linear regression model. This multivariate analysis also showed a significant negative association between *τ*_G_ and MMSE only (*p* = 0.007 for the association between *τ*_G_ and MMSE, model *r* = 0.402, model adjusted *r*^2^ = 0.121, *F* = 3.961). Similar to the univariate results, none of the parameters were significantly associated with CDR in a multivariate regression model after adjusting for multiple testing.

## 3 Discussion

The goal of this study was to employ biophysical model-based reasoning to uncover AD-related changes in the spectral characteristics of neuronal activity. We leveraged the high spatiotemporal resolution of MEG to derive characteristics of altered spectral signatures and mapped them onto mesoscopic and macroscopic parameters of a computational model of brain activity. Our chosen model was the spectral graph model (SGM), which is ideally suited for this exploration since it is linear, easy to evaluate, tractable to infer, gives power spectra directly, and most importantly, serves as a computational link between structure and function in the brain. We were able to demonstrate that global mechanisms of neural activity are significantly altered in patients with AD. Specifically, we found that the characteristic time constant associated with long-range excitatory connections is the most sensitive biophysical property that mediates altered global network dynamics in patients with AD. This parameter not only recapitulates the spectral shifts observed in AD but is also correlated with global cognitive deficits in patients with AD. This and other global network parameters were excellent computational disease biomarkers since they could accurately classify AD versus controls with a random forest classifier. The current investigation also demonstrates the ability of a model which uses global, spatially-invariant parameters, and the structural connectome as the basis for signal communication to successfully capture the spectral signatures in both degree and distribution.

To our knowledge, this is the first report of a single global parameter change that can reliably produce spectral and regional shifts from healthy to AD brains and is also correlated with cognitive deficits. Our findings provide critical insights about potential mechanistic links between abnormal neural oscillations and cellular correlates of impaired neuronal activity in AD. Because such insights from non-invasive neuroimaging data in clinical populations can only be obtained through a combination of mathematical modeling, our study fills a key missing link between impaired long-range excitatory connections and cognitive deficits in patients with AD.

### 3.1 Biophysical significance of highlighted model parameters

The parameters that were differentially distributed in AD were the excitatory time constants *τ*_G_ and *τ*_e_, and inhibitory neural gain *g*_ii_. Each parameter has a distinct biophysical meaning, and clear implications in AD pathophysiology, as discussed below.

#### Macroscopic time constant.

The most important differential parameter in our work was the long-range excitatory time constant *τ*_G_, which was substantially higher in AD, was found capable of recapitulating the spectral shift seen in AD patients, and was the most important feature in classifying AD from controls. Higher *τ*_G_ in AD indicates the slowing of long-range brain-wide communication of neural activity, implicating primarily the large layer-specific pyramidal glutamatergic neurons [29]. These pyramidal neurons are well-known to be selectively vulnerable in AD and indeed many other neurodegenerative diseases [30]. In addition, this result is in concordance with a recent study demonstrating long-range axonal connectivity disruption in AD in a mouse model [31]. Intriguingly, we report that *τ*_G_ is also associated with global cognitive deficits in patients. This implicates impairment in the synaptic processing of macroscopic long-range excitatory neurons, a potential factor in the elongation of *τ*_G_, as a key marker of AD. Our recent study showed that alpha hyposynchrony is correlated with the degree of global cognitive dysfunction in patients with AD [17]. While such associations can be obtained using neuroimaging data directly, here we were able to identify a specific biophysically grounded parameter, *τ*_G_, that can potentially explain the biological relationship. Linking biophysical processes to clinical scales has historically been extremely challenging for conventional machine learning approaches due to the mismatch in dimensionality between input features (thousands) and output features (a handful of clinical measures). Increased long-range time constant in AD, capable of recapitulating the spectral shifts in AD and correlated with MMSE, therefore, may be the first report of a single biophysical correlate accounting for clinical deficits in patients with AD.

#### Mesoscopic excitatory time constant.

The SGM model incorporates two additional neural time constants: a lumped mass of local (mesoscopic) excitatory and a mass of local inhibitory neural populations. Local excitatory-inhibitory imbalances in AD have been demonstrated in numerous basic science studies [32]. Consistent with our previous investigation [18], we also found that excitatory and inhibitory neural parameters within local ensembles are abnormal in patients with AD, although their effect sizes were small compared to the long-range time constant parameter. Among the local parameters, the strongest relationship was found with the mesoscopic excitatory time constant *τ*_e_, which was higher in AD subjects than in controls, consistent with our previous findings. While higher *τ*_e_ implies the slowing of the short-range excitatory signals at the mesoscopic level, we previously demonstrated that increased *τ*_e_ is distinctly associated with tau accumulation in AD. The relationship between spatially invariant long-range excitatory time constant and regional tau accumulation in AD remains to be elucidated.

#### Inhibitory neural gain

We also found that the inhibitory neural gain *g*_ii_ is higher in AD subjects than in controls. Neural gains incorporate the gain in internal recurrent signals due to local connectivities within a specific region. Higher *g*_ii_ implies a higher gain of the inhibitory neural signals at the mesoscopic level. Alteration in the neural gain term indicates a neuronal excitatory-inhibitory imbalance; such an imbalance has been reported in various preclinical AD models [33, 15, 34, 35]. Overall increased inhibitory gain was found in epilepsy [36], while the local subpopulation estimates in AD showed reductions as seen in our previous study [18]. This discrepancy requires further study in the future.

### 3.2 Spatially invariant network effects

Due to the highly specific spatial topography of AD pathology [6], prior literature has broadly focused on the neural correlates of local circuits as the primary means of describing observed electrophysiological data [32]. In a recent study, we implemented a local linearized neural mass model of activity, and fit it to the regional MEG spectra in AD patients. We used this model, with spatially-varying neural masses and their parameters, to test the hypothesis that local changes in model parameters could recapitulate regional spectral shifts in AD patients. We also reported profound alterations in local excitatory to inhibitory parameters that attribute to regional distributions of tau and amyloid-*β* PET imaging in AD patients [18]. The current study addresses a very different hypothesis: that observed alterations in MEG in AD patients may be explained by *global* changes in the network, as compared to spatially-varying changes in local neural masses. The SGM model used here combines local excitatory and inhibitory neural masses with a macroscopic connectome-based network of excitatory connections. Therefore in this study, we have kept all mesoscopic (local) parameters identical across the entire brain and only allowed them to change globally.

While the two hypotheses on spatially variant versus invariant effects in AD are not mutually exclusive, our key contribution here is to show that global changes are fully sufficient in their own right. A previous modeling study using coupled neural masses also found differences in both coupling and local circuits [24]. Even though AD may induce both local and global changes, it is possible that the latter may dominate, as previously noted from a modeling perspective [37–41] and from our results indicating macroscopic *τ*_G_ as the most important parameter. Heterogeneity of the Amyloid-*β* load was previously found to be essential to simulate the slowing of rhythm [42], but we have demonstrated that spatial variations of any kind in our model are not needed to capture the spectral and alpha-band spatial patterns. This certainly leaves room for the possibility that the SGM will be enriched by including the spatial patterns of Amyloid-*β* and tau. Future explorations of the respective contributions of local versus global network changes in AD will be critical.

This local versus global distinction also means that our current results are not directly comparable to prior spatially-variable modeling results. Both the macroscopic (*τ*_G_) and the mesoscopic (*τ*_e_ and *g*_ii_) parameters in our SGM model showed significant group differences, but we did not reproduce other local changes reported, e.g., in [18]. Nevertheless, *τ*_e_ being higher in AD in both the local as well as global study indicate a common underlying mechanism involving excitatory neuronal subpopulations at both local and global level in AD. Our local study had found a reduced inhibitory gain *g*_ii_ in AD patients compared to controls [18], whereas the current global study did not; possibly because the current *g*_ii_ is regionally-invariant, and was not uniquely estimated for each region’s local inhibitory subpopulation.

### 3.3 Intriguing link to eigenmodes responsible for pathology transmission

It was previously shown by our group that the eigendecomposition of the graph Laplacian can be used to describe the spread of pathology as it ramifies within the brain’s anatomic connectivity network. It was demonstrated that only the eigenmodes corresponding to the lowest eigenvalues - named “persistent modes” are involved in AD pathology progression [27]. Since any aberration in long-range synchrony explored here must arise from the underlying progression of pathology in the AD brain, it is expected that the same or similar eigenmodes responsible for pathology progression may also be involved in aberrant FC. The SGM too can be decomposed into a small set of eigenmodes (see Eq 1). Remarkably, it was recently shown by our group that the lowest few eigenmodes of the SGM capture a large portion of the spatial distribution of alpha-band power [26], and are also important in explaining low-frequency FC from BOLD fMRI [43]. This striking resemblance of eigenmodes of both pathological and electrophysiological processes establishes a conceptual bridge that has been hitherto unknown.

### 3.4 Relationship to previous modeling works

Even though no mathematical model can capture the complex brain structure-function relationship completely, many can aid in identifying mechanisms that cannot be inferred with neuroimaging data alone. Indeed, various model-based markers of AD have also been shown in the past. For example, the Virtual Brain Modeling platform has been used to estimate local and global parameters of a neural mass model for fMRI and to subsequently differentiate between AD and controls [24]. While the literature on fMRI studies in AD is vast, comparable depth is lacking in the use of higher frequency data like MEG. In our work, we focus on MEG because it provides us with a high temporal resolution and can give insights into oscillatory signatures, especially the spectral and spatial patterns thereof, that are directly linked to cellular mechanisms. A neural mass modeling approach attributed slowing of alpha in AD using MEG to neuronal hyperactivity, though without directly fitting to the empirical data [44]. Another modeling approach examined different stimulation strategies to preserve functional network integrity in AD and found that stimulating excitatory neurons were the most successful [45]. Another virtual brain simulation approach integrated local field potential simulations with regional amyloid-*β* and tau uptake as empirical features to classify healthy controls, MCI, and AD and obtained an average F1 score of 0.743 [46] – our study reports a higher F1 score of 0.77 for classification of AD from controls with just a few parameters as features of a random forest classifier.

A key difference from prior modeling approaches is that our SGM is a linear model with a small set of biophysically interpretable global parameters. Therefore, it can be obtained in a closed-form solution in the frequency domain and model parameter inference is more tractable. We employed SGM because prior studies indicate that the emergent macroscopic activity is independent of the microscopic activity of individual neurons [37–39, 47, 40, 41], and is primarily governed by the long-range connections [48–51]. Indeed, it was already demonstrated that SGM outperforms a Wilson-Cowan neural mass model in fitting the empirical MEG spectra [25]. A recent comparison showed that linear models outperformed non-linear models in predicting resting-state fMRI time series. This was attributed to the linearizing effects of macroscopic neurodynamics and neuroimaging due to spatial and temporal averaging, observation noise, and high dimensionality [52]. Given that the vast majority of computational models involving neural masses involve highly non-linear concepts like multistability, metastability, and other complex dynamics [53–57], it may be questioned whether AD-induced changes in brain macroscopic dynamics can even be reliably measured and robustly inferred. Instead, we expect that while neural activity in AD and health might be highly dynamic and non-linear, its macroscopic spatial and frequency patterns are known to be far more stable across individuals [38, 58, 59, 17, 18]. This is a key motivation for our use of the linear and deterministic SGM, which has demonstrable tractability and only a few free parameters capable of predicting spectral and regional profiles of MEG activity. To our knowledge, this is the first study identifying a parsimonious biophysically interpretable marker of AD and cognitive decline in AD.

### 3.5 Limitations of the current study

In this study, we aimed to capture the shape of the power spectra by using Pearson’s R as the cost function. In the future, we will aim at capturing the magnitude of the power spectra as well as selected spectral features. Further, we employed the same template structural connectome from HCP for both cohorts, in the interest of statistical tractability. With this, we can pinpoint the biophysical alterations solely due to alterations in the functional activity. In addition, a prior study has demonstrated that white matter network organization is preserved in AD [60]. In the future, however, we will obtain individual structural connectomes in AD. Lastly, we observed that the SGM fits better to spectral and spatial patterns from AD rather than from controls. This may be attributed to the spectral shape of AD – it has a clearer exponential fall-off that is easier to fit to. In comparison, the spectral shape of controls has an additional peak in the beta band superimposed on the exponential fall-off.

Notwithstanding, this work shows that a global impairment in the excitatory long-range pyramidal neuronal population is the most important indicator of AD, and is also associated with global cognitive decline in patients with AD. Our modeling approach outlines a parsimonious framework for identifying cellular correlates of abnormal electrophysiological oscillations and cognitive deficits in AD, that can aid in guiding future clinical trials.

## 4 Methods

### 4.1 Data description

88 patients with AD (diagnostic criteria for probable AD or mild cognitive impairment due to AD) [61–63] and 88 age-matched controls were included in this study. Each participant underwent a complete clinical history, physical examination, neuropsychological evaluation, brain magnetic resonance imaging (MRI), and a 5-10-minute session of resting MEG. All participants with AD were recruited from research cohorts at the University of California San Francisco-Alzheimer’s Disease Research Center(UCSF-ADRC). Healthy control participants were recruited at UCSF-ADRC as well as from several ongoing studies at the Biomagnetic Imaging Laboratory at UCSF. Informed consent was obtained from all participants and the study was approved by the Institutional Review Board (IRB) at UCSF (UCSF-IRB 10-02245). The mean (std) age of controls (N=88) and patients with AD (N=88) was 65.07 (9.92) and 62.73 (8.64) years, respectively. 51 (58 %) of 88 controls, and 53 (60.2 %) of patients with AD were females. The mean (std) MMSE score of patients with AD was 22.14 (5.55), while the mean Clinical Dementia Rating-Sum of Boxes (CDR) score of patients with AD was 4.90 (2.75).

### 4.2 Clinical assessments and MEG, and MRI acquisition and analyses

All the processing pipelines are the same as that for a previous study [18]. Patients with AD were assessed via MMSE and a standard battery of neuropsychological tests. Patients with AD were assessed via a structured caregiver interview to determine the Clinical Dementia Rating.

MEG scans were acquired on a whole-head biomagnetometer system (275 axial gradiometers; MISL, Coquitlam, British Columbia, Canada) for 5–10 min, following the same protocols described previously [17, 18]. Tomographic reconstructions of source-space data were done using a continuous 60-second data epoch, an individualized head model based on structural MRI, and a frequency optimized adaptive spatial filtering technique implemented in the Neurodynamic Utility Toolbox for MEG (NUTMEG; http://nutmeg.berkeley.edu). We derived the regional power spectra based on Desikan–Killiany atlas parcellations for the 68 cortical regions depicting neocortex and allocortex, the latter including the entorhinal cortex. Regional power spectra were derived from FFT and then converted to dB scale.

### 4.3 Resting state MEG data acquisition

Each subject underwent MEG recording on a whole-head biomagnetometer system consisting of 275 axial gradiometers (MISL, Coquitlam, British Columbia, Canada), for 5–10 min. Three fiducial coils including nasion, left and right preauricular points were placed to localize the position of head relative to sensor array, and later coregistered to each individual’s respective MRI to generate an individualized head shape. Data collection was optimized to minimize within-session head movements and to keep it below 0.5 cm. 5–10 min of continuous recording was collected from each subject while lying supine and awake with eyes closed (sampling rate: 600 Hz). We selected a 60-s (1 min) continuous segment with minimal artifacts (minimal excessive scatter at signal amplitude <10 pT), for each subject, for analysis. The study protocol required the participant to be interactive with the investigator and be awake at the beginning of the data collection. Spectral analysis of each MEG recording and whenever available, and the simultaneously collected scalp EEG recording were examined to confirm that the 60-s data epoch represented awake, eyes closed resting state for each participant. Artifact detection was confirmed by visual inspection of sensor data and channels with excessive noise within individual subjects were removed prior to analysis.

### 4.4 Source space reconstruction of MEG data and spectral power estimation

Tomographic reconstructions of the MEG data were generated using a head model based on each participant’s structural MRI. Spatiotemporal estimates of neural sources were generated using a time-frequency optimized adaptive spatial filtering technique implemented in the Neurodynamic Utility Toolbox for MEG (NUTMEG; https://nutmeg.berkeley.edu/). Tomographic volume of source locations (voxels) was computed through an adaptive spatial filter (10-mm lead field) that weights each location relative to the signal of the MEG sensors [64, 65]. The source space reconstruction approach provided amplitude estimations at each voxel derived through the linear combination of spatial weighting matrix with the sensor data matrix [64]. A high-resolution anatomical MRI was obtained for each subject (see below) and was spatially normalized to the Montreal Neurological Institute (MNI) template brain using the SPM software (http://www.fil.ion.ucl.ac.uk/spm), with the resulting parameters being applied to each individual subject’s source space reconstruction within the NUTMEG pipeline [65].

To prepare for source localization, all MEG sensor locations were coregistered to each subject’s anatomical MRI scans. The lead field (forward model) for each subject was calculated in NUTMEG using a multiple local-spheres head model (three-orientation lead field) and an 8-mm voxel grid which generated more than 5000 dipole sources, all sources were normalized to have a norm of 1. The MEG recordings were projected into source space using a beamformer spatial filter. Source estimates tend to have a bias towards superficial currents and the estimates are more error-prone when we approach subcortical regions, therefore, only the sources belonging to the 68 cortical regions were selected for further analyses. Specifically, all dipole sources were labeled based on the Desikan–Killiany parcellations, then sources within a 10-mm radial distance to the centroid of each brain region were extracted for each region. In this study, we examined the broad-band (1–35 Hz). Power spectra were derived by applying FFT on the time-course data and then converted to the dB scale.

### 4.5 Magnetic resonance image acquisition and analysis

Structural brain images were acquired from all participants using a unified MRI protocol on a 3 Tesla Siemens MRI scanner at the Neuroscience Imaging Center (NIC) at UCSF. Structural MRIs were used to generate individualized head models for source space reconstruction of MEG sensor data. Structural MRI scans were also used in the clinical evaluations of patients with AD to identify the pattern of gray matter volume loss to support the diagnosis of AD.

### 4.6 Model

The model used here is similar to the SGM developed previously [25, 26, 66, 67], and is described in detail in the supplementary document. Briefly, it is characterized by the following model parameters at the mesoscopic level: excitatory time constant (*τ*_e_), inhibitory time constant (*τ*_i_), excitatory gain (*g*_ee_, assumed to be 1 for parameter identifiability), inhibitory gain (*g*_ii_), coupled population gain (*g*_ei_); and the following model parameters at the macroscopic level: coupling constant (*α*), speed (*v*), graph excitatory time constant (*τ*_G_). The model solution can be obtained in a closed form in the frequency domain as a function of angular frequency *ω* as:

(1) $$X(\omega )=\overset{N}{\underset{k=1}{\sum }}\frac{u_{k}(\omega )u_{k}{\left(\omega \right)}^{\text{H}}}{\text{j}\omega +\tau_{\text{G}}^{-1}\lambda_{k}(\omega )F_{\text{G}}(\omega )}H_{\text{local}}(\omega )P(\omega ),$$

where, ***X***(*ω*) is the signal of every brain region, *u_k_*(*ω*) are the eigenmodes and *λ*_*k*_(*ω*) are the eigenvalues obtained by the eigen-decomposition of a complex Laplacian matrix. Equation (1) is the closed-form steady- state solution of the macroscopic signals at a specific angular frequency *ω*. We use this modeled spectra to compare against empirical MEG spectra and subsequently estimate model parameters. In practice, only a few eigenmodes *k* ∈ [1, *K*], *K* ≪ *N* are needed to obtain sufficiently strong fits to empirical data, including especially the lowest eigenmodes [26].

### 4.7 Model parameter estimation

The model parameter estimation procedure is same as described previously [67]. Modeled spectra was converted into PSD by calculating the norm of the frequency response and converting it to dB scale by taking 20log_10_() of the norm. Pearson’s *r* between modeled PSD and the MEG PSD was used a goodness of fit metric for estimating model parameters. Pearson’s *r* between modeled and MEG PSD was computed for all 68 brain regions. Its average *r* across all regions is referred to as the *spectral correlation*. Next we calculated the *spatial correlation* by obtaining the regional distribution of alpha band (8-12 Hz) raw power of both model ***x*** and MEG ***y***. Then, the spatial correlation was defined as ***x***^*T*^ ‖(***C*** + *w**I***)‖ ***y***, where ***C*** is the row degree normalized structural connectivity matrix, ***I*** is the identity matrix, *w* is an empirical weight, and ‖(***C*** + 10***I***)‖ is the row normalized version of ***C*** + 10***I***. The objective function for optimization and estimation of model parameters was the sum of spectral and spatial correlations. We used a dual annealing optimization procedure in Python for performing parameter optimization [68].

Parameter initial guesses and bounds for estimating the static spectra are specified in Table 1. We defined three different bounds on the neural gain terms to ensure that the model is stable, based on prior work on model stability [66]. First, we supplied a larger bound on the neural gains for optimization. If the optimal model parameter was outside the stability boundary, we repeated optimization with a smaller bound. We repeated this procedure 3 times to ensure that the final optimal model parameters correspond to the stable model solutions. We used a dual annealing optimization procedure in Python for parameter optimization [68]. The dual annealing optimization was performed for three different initial guesses, and the parameter set leading to maximum sum of spectral and spatial correlations was chosen for each subject. The dual annealing settings were: maxiter = 500. All the other settings were the same as default.

### 4.8 Statistical analyses

Statistical tests were performed using SAS software (SAS9.4; SAS Institute, Cary, NC) and the statsmodels package in Python. To compare the neuronal parameters between the controls and patients, we used a linear mixed-effects model (PROC MIXED), to compare model parameters (*τ*_G_, *τ*_e_, *τ*_i_, *g*_ii_, *g*_ei_, *α*, *v*), including age as a covariate into the models. We reported the estimated least-squares means and the statistical differences of least-squares means based on unpaired t-tests. We also developed univariate linear regression models to examine the associations between model parameters and MMSE and CDR scores in AD. In these models, the dependent variables included MMSE and CDR (in separate models), and the predictor variables included the model parameters we found significant between AD and controls (*τ*_G_, *τ*_e_, and *g*_ii_). Next, we developed multivariate linear regression models with dependent variables as MMSE and CDR (separately), and the predictor variables included all the significant parameters *τ*_G_ *τ*_e_, *g*_ii_, and age as covariates.

### 4.9 Classification between AD and controls

We trained a random forest for classifying AD and controls. Here, we used the SGM parameters and age as features of the model. For training and testing, we employed a 5-fold stratified cross validation method. We divided the dataset into 5 folds and used the 4 folds for training, and the 5^th^ fold for testing the model. We repeated this procedure 100 times. While training the model, no information of the testing fold was provided. With the 4 folds of the training dataset, further 5-fold cross validation was performed to estimate the tuning parameter of the random forest. Here, we only tuned for the max depth with the following options for max depth: None, 2, 3, 4. All other hyperparameters were kept as default in the sklearn package in Python. After estimating the tuning parameter, the model was trained using the entire training dataset and then tested on the 5^th^ fold. The mean AUROC of the test dataset was finally reported. The feature importance was estimated as the average of the feature importance from the random forest classifier that was trained 100 times.

## Figures and Tables

**Table 1:** SGM parameter values, initial guesses, and bounds for parameter estimation for static spectra fitting

Name	Symbol	Initial value 1	Initial value 2	Initial value 3	Lower/upper bound for optimization
Excitatory time constant	*τ_e_*	0.015 s	0.025 s	0.006 s	[0.005 s, 0.03 s]
Inhibitory time constant	*τ_i_*	0.01 s	0.08 s	0.15 s	[0.005 s, 0.2 s]
Long-range connectivity coupling constant	*α*	1	0.5	0.1	[0.1, 1]
Transmission speed	*v*	5 m/s	10 m/s	18 m/s	[5 m/s, 20 m/s]
Alternating population gain	*g_ei_*	0.3	0.2	0.1	[0.001,0.7], [0.001,0.5], [0.001,0.4]
Inhibitory gain	*g_ii_*	0.6	0.1	1.2	[0.001,2.0], [0.001,1.5], [0.001,1.5]
Graph time constant	*τ_G_*	0.006 s	0.015 s	0.025 s	[0.005 s, 0.03 s]
Excitatory gain	*g_ee_*	n/a	n/a	n/a	n/a

## Data Availability

The code for this work is available as an anonymized repository here: https://anonymous.4open.science/r/sgm-ad-5C35. This will be made publicly available once the manuscript is made public. Anonymized subject data will be shared on request from qualified investigators for the purposes of replicating procedures and results, and for other non-commercial research purposes within the limits of participants’ consent. Correspondence and material requests should be addressed to Kamalini.ranasinghe@ucsf.edu.

## References

[1] Babiloni Claudio, Lizio Roberta, Marzano Nicola, Capotosto Paolo, Soricelli Andrea, Antonio Ivano Triggiani Susanna Cordone, Gesualdo Loreto, and Claudio Del Percio. Brain neural synchronization and functional coupling in Alzheimer’s disease as revealed by resting state EEG rhythms. International Journal of Psychophysiology, 103:88–102, 2016. 25660305 10.1016/j.ijpsycho.2015.02.008

[2] Wang Ruofan, Wang Jiang, Yu Haitao, Wei Xile, Yang Chen, and Deng Bin. Power spectral density and coherence analysis of Alzheimer’s EEG. Cognitive Neurodynamics, 9(3):291–304, 2015. 25972978 10.1007/s11571-014-9325-x PMC4427585

[3] Sedghizadeh Mohammad, Aghajan Hamid, Zahra Vahabi, Fatemi Seyyedeh, and Afzal Arshia. Network synchronization deficits caused by dementia and Alzheimer’s disease serve as topographical biomarkers: a pilot study. Brain Structure and Function, 227:2957–2969, 2022. 35997832 10.1007/s00429-022-02554-2 PMC9396580

[4] Sitnikova Tatiana A., Hughes Jeremy W., Ahlfors Seppo P., Woolrich Mark W., and Salat David H.. Short timescale abnormalities in the states of spontaneous synchrony in the functional neural networks in alzheimer’s disease. NeuroImage: Clinical, 20:128–152, 2018. 30094163 10.1016/j.nicl.2018.05.028 PMC6077178

[5] Ogawa Seiji, Lee Tso-Ming, Alan R Kay, and David W Tank. Brain magnetic resonance imaging with contrast dependent on blood oxygenation. proceedings of the National Academy of Sciences, 87(24):9868–9872, 1990. 10.1073/pnas.87.24.9868 PMC55275 2124706

[6] Jagust William. Imaging the evolution and pathophysiology of alzheimer disease. Nature Reviews Neuroscience, 19(11):687–700, 2018. 30266970 10.1038/s41583-018-0067-3 PMC7032048

[7] Supekar Kaustubh, Menon Vinod, Rubin Daniel, Musen Mark, and Michael D Greicius. Network analysis of intrinsic functional brain connectivity in alzheimer’s disease. PLoS computational biology, 4(6):e1000100, 2008. 18584043 10.1371/journal.pcbi.1000100 PMC2435273

[8] Sanz-Arigita Ernesto J, Schoonheim Menno M, Damoiseaux Jessica S, Rombouts Serge ARB, Maris Erik, Frederik Barkhof, Philip Scheltens, and Cornelis J Stam. Loss of ‘small-world’networks in alzheimer’s disease: graph analysis of fmri resting-state functional connectivity. PloS one, 5(11):e13788, 2010. 21072180 10.1371/journal.pone.0013788 PMC2967467

[9] Parker Andrew, Derrington Andrew, Blakemore Colin, and Logothetis Nikos K.. The neural basis of the blood–oxygen–level–dependent functional magnetic resonance imaging signal. Philosophical Transactions of the Royal Society of London. Series B: Biological Sciences, 357(1424):1003–1037, 2002. 10.1098/rstb.2002.1114 PMC1693017 12217171

[10] Fernandez Alberto, Hornero Roberto, Mayo Agustin, Poza Jesus, Pedro Gil-Gregorio, and Tomas Ortiz. Meg spectral profile in alzheimer’s disease and mild cognitive impairment. Clinical Neurophysiology, 117(2):306–314, 2006. 16386951 10.1016/j.clinph.2005.10.017

[11] Stam CJ, De Haan W, Daffertshofer ABFJ, Jones BF, Manshanden I, van Cappellen van Walsum Anne-Marie, Montez Teresa, Verbunt JPA, De Munck JC, Van Dijk BW, Graph theoretical analysis of magnetoencephalographic functional connectivity in alzheimer’s disease. Brain, 132(1):213–224, 2009. 18952674 10.1093/brain/awn262

[12] Bajo Ricardo, Castellanos Nazareth P, Cuesta Pablo, Aurtenetxe Sara, Garcia-Prieto Juan, Gregorio Pedro Gil, del Pozo Francisco, and Maestu Fernando. Differential patterns of connectivity in progressive mild cognitive impairment. Brain connectivity, 2(1):21–24, 2012. 22458376 10.1089/brain.2011.0069

[13] Ranasinghe Kamalini G., Hinkley Leighton B., Beagle Alexander J., Mizuiri Danielle, Dowling Anne F., Honma Susanne M., Finucane Mariel M., Scherling Carole, Miller Bruce L., Nagarajan Srikantan S., and Vossel Keith A.. Regional functional connectivity predicts distinct cognitive impairments in alzheimer’s disease spectrum. NeuroImage: Clinical, 5:385–395, 2014. 25180158 10.1016/j.nicl.2014.07.006 PMC4145532

[14] Nakamura Akinori, Cuesta Pablo, Fernández Alberto, Arahata Yutaka, Iwata Kaori, Kuratsubo Izumi, Bundo Masahiko, Hattori Hideyuki, Sakurai Takashi, Electromagnetic signatures of the preclinical and prodromal stages of Alzheimer’s disease. Brain, 141(5):1470–1485, 03 2018. 29522156 10.1093/brain/awy044 PMC5920328

[15] Maestú Fernando, Cuesta Pablo, Hasan Omar, Fernández Alberto, Funke Michael, and Schulz Paul E.. The importance of the validation of m/eeg with current biomarkers in alzheimer’s disease. Frontiers in Human Neuroscience, 13, 2019. 10.3389/fnhum.2019.00081 PMC6415698 30894809

[16] Babiloni Claudio, Blinowska Katarzyna, Bonanni Laura, Cichocki Andrej, De Haan Willem, Del Percio Claudio, Dubois Bruno, Escudero Javier, What electrophysiology tells us about alzheimer’s disease: a window into the synchronization and connectivity of brain neurons. Neurobiology of Aging, 85:58–73, 2020. 31739167 10.1016/j.neurobiolaging.2019.09.008

[17] Ranasinghe Kamalini G, Cha Jungho, Iaccarino Leonardo, Hinkley Leighton B, Beagle Alexander J, Pham Julie, Jagust William J, Miller Bruce L, Rankin Katherine P, Rabinovici Gil D, Neurophysiological signatures in alzheimer’s disease are distinctly associated with TAU, amyloid-*β* accumulation, and cognitive decline. Science Translational Medicine, 12(534):eaaz4069, 2020. 32161102 10.1126/scitranslmed.aaz4069 PMC7138514

[18] Ranasinghe Kamalini, Verma Parul, Cai Chang, Xie Xihe, Kudo Kiwamu, Gao Xiao, Lerner Hannah, Mizuiri Danielle, Strom Amelia, Iaccarino Leonardo, Joie Renaud La, Miller Bruce L, Gorno-Tempini Maria Luisa, Rankin Katherine P, Jagust William J, Vossel Keith, Rabinovici Gil, Raj Ashish, and Nagarajan Srikantan. Altered excitatory and inhibitory neuronal subpopulation parameters are distinctly associated with tau and amyloid in alzheimer’s disease. eLife, 11:e77850, may 2022. 35616532 10.7554/eLife.77850 PMC9217132

[19] Vecchio Fabrizio, Miraglia Francesca, Curcio Giuseppe, Altavilla Riccardo, Scrascia Federica, Giambattistelli Federica, Quattrocchi Carlo, Bramanti Placido, Vernieri Fabrizio, and Rossini Paolo. Cortical brain connectivity evaluated by graph theory in dementia: a correlation study between functional and structural data. Journal of Alzheimers Disease, 45(3):745–756, 2015. 10.3233/JAD-142484 25613102

[20] Buzsaki Gyorgy. Rhythms of the Brain. Oxford university press, 2006.

[21] Gelman Simon, Palma Jonathan, and Ghavami Afshin. Axonal conduction velocity in ca1 area of hippocampus is reduced in mouse models of alzheimer’s disease. Journal of Alzheimer’s Disease, 77(4):1383–1388, 2020. 10.3233/JAD-200661 32925062

[22] Maia Pedro D and Kutz J Nathan. Compromised axonal functionality after neurodegeneration, concussion and/or traumatic brain injury. Journal of computational neuroscience, 37(2):317–332, 2014. 24916135 10.1007/s10827-014-0504-x

[23] Jones David T., Knopman David S., Gunter Jeffrey L., Jonathan Graff-Radford Prashanthi Vemuri, Boeve Bradley F., Petersen Ronald C., Cascading network failure across the Alzheimer’s disease spectrum. Brain, 139(2):547–562, 11 2015. 26586695 10.1093/brain/awv338 PMC4805086

[24] Zimmermann J., Perry A., Breakspear M., Schirner M., Sachdev P., Wen W., Kochan N. A., Mapstone M., Ritter P., McIntosh A. R., and Solodkin A.. Differentiation of Alzheimer’s disease based on local and global parameters in personalized Virtual Brain models. NeuroImage: Clinical, 19:240–251, 2018. 30035018 10.1016/j.nicl.2018.04.017 PMC6051478

[25] Raj Ashish, Cai Chang, Xie Xihe, Palacios Eva, Owen Julia, Mukherjee Pratik, and Nagarajan Srikantan. Spectral graph theory of brain oscillations. Human Brain Mapping, 41(11):2980–2998, 2020. 32202027 10.1002/hbm.24991 PMC7336150

[26] Verma Parul, Nagarajan Srikantan, and Raj Ashish. Spectral graph theory of brain oscillations—revisited and improved. NeuroImage, 249:118919, 2022. 35051584 10.1016/j.neuroimage.2022.118919 PMC9506601

[27] Raj Ashish, Kuceyeski Amy, and Weiner Michael. A network diffusion model of disease progression in dementia. Neuron, 73(6):1204–1215, 2012. 22445347 10.1016/j.neuron.2011.12.040 PMC3623298

[28] Desikan Rahul S, Ségonne Florent, Fischl Bruce, Quinn Brian T, Dickerson Bradford C, Blacker Deborah, Buckner Randy L, Dale Anders M, Maguire R Paul, Hyman Bradley T, An automated labeling system for subdividing the human cerebral cortex on MRI scans into gyral based regions of interest. Neuroimage, 31(3):968–980, 2006. 16530430 10.1016/j.neuroimage.2006.01.021

[29] Brown Solange P and Hestrin Shaul. Intracortical circuits of pyramidal neurons reflect their long-range axonal targets. Nature, 457(7233):1133–1136, 2009. 19151698 10.1038/nature07658 PMC2727746

[30] Luebke Jennifer I, Weaver Christina M, Rocher Anne B, Rodriguez Alfredo, Crimins Johanna L, Dickstein Dara L, Wearne Susan L, and Hof Patrick R. Dendritic vulnerability in neurodegenerative disease: insights from analyses of cortical pyramidal neurons in transgenic mouse models. Brain Structure and Function, 214(2):181–199, 2010. 20177698 10.1007/s00429-010-0244-2 PMC3045830

[31] Yuan Peng, Zhang Mengyang, Tong Lei, Morse Thomas M, McDougal Robert A, Ding Hui, Chan Diane, Cai Yifei, and Grutzendler Jaime. Pld3 affects axonal spheroids and network defects in alzheimer’s disease. Nature, pages 1–10, 2022. 10.1038/s41586-022-05491-6 PMC9729106 36450991

[32] Varela Eva Vico, Etter Guillaume, and Williams Sylvain. Excitatory-inhibitory imbalance in alzheimer’s disease and therapeutic significance. Neurobiology of Disease, 127:605–615, 2019. 30999010 10.1016/j.nbd.2019.04.010

[33] Palop Jorge J A network dysfunction perspective on neurodegenerative diseases. Nature, 443(7113):768–773, 2006. 17051202 10.1038/nature05289

[34] Harris Samuel S. Tipping the scales: Peptide-dependent dysregulation of neural circuit dynamics in alzheimer’s disease. Neuron, 107(3):417–435, 2020. 32579881 10.1016/j.neuron.2020.06.005

[35] Chang Siyuan, Wang Jiang, Liu Chen, Yi Guosheng, Lu Meili, Che Yanqiu, and Wei Xile. A data driven experimental system for individualized brain stimulation design and validation. IEEE Transactions on Neural Systems and Rehabilitation Engineering, 29:1848–1857, 2021. 34478377 10.1109/TNSRE.2021.3110275

[36] Elahian Bahareh, Lado Nathan E., Mankin Emily, Vangala Sitaram, Misra Amrit, Moxon Karen, Fried Itzhak, Sharan Ashwini, Yeasin Mohammed, Staba Richard, Bragin Anatol, Avoli Massimo, Sperling Michael R., Engel Jerome Jr, and Weiss Shennan A.. Low-voltage fast seizures in humans begin with increased interneuron firing. Annals of Neurology, 84(4):588–600, 2018. 30179277 10.1002/ana.25325 PMC6814155

[37] Shimizu H. and Haken H.. Co-operative dynamics in organelles. Journal of Theoretical Biology, 104(2):261–273, 1983. 6227776 10.1016/0022-5193(83)90414-9

[38] Robinson Peter A, Rennie CJ, Rowe Donald L, O’Connor SC, and Gordon E. Multiscale brain modelling. Philosophical Transactions of the Royal Society B: Biological Sciences, 360(1457):1043–1050, 2005. 10.1098/rstb.2005.1638 PMC1854922 16087447

[39] Destexhe Alain and Sejnowski Terrence J. The Wilson–Cowan model, 36 years later. Biological cybernetics, 101(1):1–2, 2009. 19662434 10.1007/s00422-009-0328-3 PMC2866289

[40] Mišić Bratislav, Sporns Olaf, and McIntosh Anthony R. Communication efficiency and congestion of signal traffic in large-scale brain networks. PLoS Comput Biol, 10(1):e1003427, 2014. 24415931 10.1371/journal.pcbi.1003427 PMC3886893

[41] Misic Bratislav, Richard F Betzel Azadeh Nematzadeh, Goni Joaquin, Griffa Alessandra, Hagmann Patric, Flammini Alessandro, Ahn Yong-Yeol, and Sporns Olaf. Cooperative and competitive spreading dynamics on the human connectome. Neuron, 86(6):1518–1529, 2015. 26087168 10.1016/j.neuron.2015.05.035

[42] Stefanovski Leon, Triebkorn Paul, Spiegler Andreas, Diaz-Cortes Margarita-Arimatea, Solodkin Ana, Jirsa Viktor, McIntosh Anthony Randal, Linking molecular pathways and large-scale computational modeling to assess candidate disease mechanisms and pharmacodynamics in alzheimer’s disease. Frontiers in computational neuroscience, page 54, 2019. 31456676 10.3389/fncom.2019.00054 PMC6700386

[43] Xie Chang Cai, Damasceno Pablo F., Nagarajan Srikantan S., and Raj Ashish. Emergence of canonical functional networks from the structural connectome. NeuroImage, 237:118190, 2021. 34022382 10.1016/j.neuroimage.2021.118190 PMC8451304

[44] van Nifterick Anne M, Gouw Alida A, van Kesteren Ronald E, Scheltens Philip, Stam Cornelis J, and de Haan Willem. A multiscale brain network model links alzheimer’s disease-mediated neuronal hyperactivity to large-scale oscillatory slowing. Alzheimer’s research & therapy, 14(1):1–20, 2022. 10.1186/s13195-022-01041-4 PMC9310500 35879779

[45] de Haan Willem, van Straaten Elisabeth C. W, Gouw Alida A, and Stam Cornelis J.. Altering neuronal excitability to preserve network connectivity in a computational model of alzheimer’s disease. PLOS Computational Biology, 13(9):1–23, 09 2017. 10.1371/journal.pcbi.1005707 PMC5627940 28938009

[46] Triebkorn Paul, Stefanovski Leon, Dhindsa Kiret, Diaz-Cortes Margarita-Arimatea, Bey Patrik, Bulau Konstantin, Pai Roopa, Spiegler Andreas, Solodkin Ana, Jirsa Viktor, Brain simulation augments machine-learning-based classification of dementia. Alzheimer’s & Dementia: Translational Research & Clinical interventions, 8(1):e12303, 2022. 35601598 10.1002/trc2.12303 PMC9107774

[47] Abdelnour Farras, Voss Henning U., and Raj Ashish. Network diffusion accurately models the relationship between structural and functional brain connectivity networks. NeuroImage, 90:335–347, 2014. 24384152 10.1016/j.neuroimage.2013.12.039 PMC3951650

[48] Jirsa V.K., Jantzen K.J., Fuchs A., and Kelso J.A.S.. Spatiotemporal forward solution of the eeg and meg using network modeling. IEEE Transactions on Medical Imaging, 21(5):493–504, 2002. 12071620 10.1109/TMI.2002.1009385

[49] Deco Gustavo, Senden Mario, and Jirsa Viktor. How anatomy shapes dynamics: a semi-analytical study of the brain at rest by a simple spin model. Frontiers in Computational Neuroscience, 6:68, 2012. 23024632 10.3389/fncom.2012.00068 PMC3447303

[50] Nakagawa Tristan T., Woolrich Mark, Luckhoo Henry, Joensson Morten, Mohseni Hamid, Kringelbach Morten L., Jirsa Viktor, and Deco Gustavo. How delays matter in an oscillatory whole-brain spiking-neuron network model for MEG alpha-rhythms at rest. NeuroImage, 87:383–394, 2014. 24246492 10.1016/j.neuroimage.2013.11.009

[51] Abdelnour F, Raj A, Dayan M, Devinsky O, and Thesen T. Estimating function from structure in epileptics using graph diffusion model. In 2015 IEEE 12th international Symposium on Biomedical Imaging (ISBI), pages 466–469, 2015.

[52] Nozari Erfan, Stiso Jennifer, Caciagli Lorenzo, Cornblath Eli J., He Xiaosong, Bertolero Maxwell A., Mahadevan Arun S., Pappas George J., and Bassett Danielle S.. Is the brain macroscopically linear? a system identification of resting state dynamics. bioRxiv, 2020.

[53] Freyer Frank, Roberts James A., Becker Robert, Robinson Peter A., Ritter Petra, and Breakspear Michael. Biophysical Mechanisms of Multistability in Resting-State Cortical Rhythms. Journal of Neuroscience, 31(17):6353–6361, 2011. 21525275 10.1523/JNEUROSCI.6693-10.2011 PMC6622680

[54] Deco Gustavo and Jirsa Viktor K.. Ongoing Cortical Activity at Rest: Criticality, Multistability, and Ghost Attractors. Journal of Neuroscience, 32(10):3366–3375, 2012. 22399758 10.1523/JNEUROSCI.2523-11.2012 PMC6621046

[55] Cabral Joana, Kringelbach Morten L., and Deco Gustavo. Exploring the network dynamics underlying brain activity during rest. Progress in Neurobiology, 114:102–131, 2014. 24389385 10.1016/j.pneurobio.2013.12.005

[56] Golos Mathieu, Jirsa Viktor, and Daucé Emmanuel. Multistability in Large Scale Models of Brain Activity. PLOS Computational Biology, 11(12):1–32, 12 2016. 10.1371/journal.pcbi.1004644 PMC4692486 26709852

[57] Deco Gustavo, Kringelbach Morten L, Jirsa Viktor K, and Ritter Petra. The dynamics of resting fluctuations in the brain: metastability and its dynamical cortical core. Scientific reports, 7(1):1–14, 2017. 28596608 10.1038/s41598-017-03073-5 PMC5465179

[58] Freeman Walter J and Zhai Jian. Simulated power spectral density (PSD) of background electrocor-ticogram (ECoG). Cognitive neurodynamics, 3(1):97–103, 2009. 19003455 10.1007/s11571-008-9064-y PMC2645494

[59] He Biyu J., Zempel John M., Snyder Abraham Z., and Raichle Marcus E.. The Temporal Structures and Functional Significance of Scale-free Brain Activity. Neuron, 66(3):353–369, 2010. 20471349 10.1016/j.neuron.2010.04.020 PMC2878725

[60] Powell Fon, Tosun Duygu, Sadeghi Roksana, Weiner Michael, Raj Ashish, Alzheimer’s Disease Neuroimaging Initiative, Preserved structural network organization mediates pathology spread in alzheimer’s disease spectrum despite loss of white matter tract integrity. Journal of Alzheimer’s Disease, 65(3):747–764, 2018. 10.3233/JAD-170798 PMC6152926 29578480

[61] Albert Marilyn S., DeKosky Steven T., Dickson Dennis, Dubois Bruno, Feldman Howard H., Fox Nick C., Gamst Anthony, Holtzman David M., The diagnosis of mild cognitive impairment due to alzheimer’s disease: Recommendations from the national institute on aging-alzheimer’s association workgroups on diagnostic guidelines for alzheimer’s disease. Alzheimer’s & Dementia, 7(3):270–279, 2011. 10.1016/j.jalz.2011.03.008 PMC3312027 21514249

[62] McKhann Guy M., Knopman David S., Chertkow Howard, Hyman Bradley T., Jack Clifford R. Jr, Kawas Claudia H., Klunk William E., The diagnosis of dementia due to alzheimer’s disease: Recommendations from the national institute on aging-alzheimer’s association workgroups on diagnostic guidelines for alzheimer’s disease. Alzheimer’s & Dementia, 7(3):263–269, 2011. 10.1016/j.jalz.2011.03.005 PMC3312024 21514250

[63] Jack Clifford R. Jr., Bennett David A., Blennow Kaj, Carrillo Maria C., Dunn Billy, Haeberlein Samantha Budd, Holtzman David M., Jagust William, Nia-aa research framework: Toward a biological definition of alzheimer’s disease. Alzheimer’s & Dementia, 14(4):535–562, 2018. 10.1016/j.jalz.2018.02.018 PMC5958625 29653606

[64] Dalal Sarang S., Guggisberg Adrian G., Edwards Erik, Sekihara Kensuke, Findlay Anne M., Canolty Ryan T., Berger Mitchel S., Knight Robert T., Barbaro Nicholas M., Kirsch Heidi E., and Nagarajan Srikantan S.. Five-dimensional neuroimaging: Localization of the time–frequency dynamics of cortical activity. NeuroImage, 40(4):1686–1700, 2008. 18356081 10.1016/j.neuroimage.2008.01.023 PMC2426929

[65] Dalal Sarang S, Zumer Johanna M, Guggisberg Adrian G, Trumpis Michael, Wong Daniel DE, Sekihara Kensuke, and Nagarajan Srikantan S. Meg/eeg source reconstruction, statistical evaluation, and visualization with nutmeg. Computational intelligence and neuroscience, 2011, 2011. 10.1155/2011/758973 PMC3061455 21437174

[66] Verma Parul, Nagarajan Srikantan, and Raj Ashish. Stability and dynamics of a spectral graph model of brain oscillations. Network Neuroscience, pages 1–43, 07 2022. 37334000 10.1162/netn_a_00263 PMC10270709

[67] Raj Ashish, Verma Parul, and Nagarajan Srikantan. Structure-function models of temporal, spatial, and spectral characteristics of non-invasive whole brain functional imaging. Frontiers in neuroscience, 16:959557–959557, 2022. 36110093 10.3389/fnins.2022.959557 PMC9468900

[68] Xiang Y, Sun D.Y, Fan W, and Gong X.G. Generalized simulated annealing algorithm and its application to the Thomson model. Physics Letters A, 233(3):216–220, 1997.

