# Impaired long-range excitatory time scale predicts abnormal neural oscillations and cognitive deficits in Alzheimer’s disease

**DOI:** 10.21203/rs.3.rs-2579392/v3

**Published:** 2023-11-01

**Authors:** Parul Verma, Kamalini Ranasinghe, Janani Prasad, Chang Cai, Xihe Xie, Hannah Lerner, Danielle Mizuiri, Bruce Miller, Katherine Rankin, Keith Vossel, Steven W. Cheung, Srikantan Nagarajan, Ashish Raj

**Affiliations:** 1 Department of Radiology and Biomedical Imaging, University of California San Francisco, San Francisco, CA, USA; 2 Memory and Aging Center, Department of Neurology, University of California San Francisco, San Francisco, CA, USA; 3 Amador Valley High School, Pleasanton, CA, USA; 4 Mary S. Easton Center for Alzheimer’s Research and Care, Department of Neurology, David Geffen School of Medicine, University of California Los Angeles, Los Angeles, CA, USA; 5 Department of Otolaryngology-Head and Neck Surgery, University of California San Francisco, San Francisco, CA, USA; 6 Surgical Services, Veterans Affairs, San Francisco, USA

**Keywords:** brain activity, Alzheimer’s disease, magnetoencephalography, spectral graph theory, cognitive decline

## Abstract

Alzheimer’s disease (AD) is the most common form of dementia, progressively impairing memory and cognition. While neuroimaging studies have revealed functional abnormalities in AD, how these relate to aberrant neuronal circuit mechanisms remains unclear. Using magnetoencephalography imaging we documented abnormal local neural synchrony patterns in patients with AD. To identify abnormal biophysical mechanisms underlying these abnormal electrophysiological patterns, we estimated the parameters of a spectral graph-theory model (SGM). SGM is an analytic model that describes how long-range fiber projections in the brain mediate the excitatory and inhibitory activity of local neuronal subpopulations. The long-range excitatory time scale was associated with greater deficits in global cognition and was able to distinguish AD patients from controls with high accuracy. These results demonstrate that long-range excitatory time scale of neuronal activity, despite being a global measure, is a key determinant in the spatiospectral signatures and cognition in AD.

## 1 Introduction

Alzheimer’s disease (AD) is the most common form of dementia, progressively impairing the cognition and behavior of the affected individual. It has been proposed that the effect of AD neurodegeneration on cortical neuronal networks is partially reflected by the abnormal mechanisms of cortical neural synchronization and coupling [1]. Neural synchronization refers to the simultaneous activity of neuronal groups in the brain. Repetitive spiking activities of neural populations manifests as oscillations ranging from slow delta to fast gamma frequencies. Synchronization of neural oscillations may represent both *local synchrony*, typically estimated from regional level power spectral density (PSD) of the electrophysiological signal, and *long-range synchrony*, estimated from pair-wise coherences between signals originating at different locations. Neurodegeneration disrupts both local and long-range synchrony [2, 3]. Because functional deficits precede the structural deficits in AD, it is likely that local and long-range synchrony deficits occur before the onset of clinical symptoms, may worsen as the disease progresses, and may even play a role in disease manifestation [4, 5]. It is therefore important to understand how synchrony within and between brain regions is disrupted in AD and is associated with cognitive impairment.

Electro- or magneto-encephalography (E/MEG), that capture temporal functional activity scales with millisecond precision, studies have shown thats both local and long-range synchrony are abnormal in AD [6, 7].The relative PSD of patients with AD is significantly increased in the delta and the theta, while reduced in the alpha frequency band [8–12, 2, 13], often referred to as an oscillatory slowing. This oscillatory slowing has also been observed in MEG studies by our group and others [14–18, 6, 19–21]. Interestingly, these aberrant local synchrony patterns are associated with the pathological processes in AD [22]. These data have naturally led to a search for a common underlying neural mechanism whose impairment might account for observed spatial and spectral shifts in E/MEG imaging data in AD patients.

In this paper, we hypothesize that global alterations in neural mechanisms for synchrony can account for the abnormal spatiospectral profile of neural oscillations across the brain as observed in MEG, and predict cognitive decline in patients with AD. We test this hypothesis using a biophysical model of whole-network level brain activity, called the Spectral Graph Model (SGM), which can capture these phenomena with a parsimonious set of biophysically interpretable parameters. SGM posits that the anatomical network of fiber projections is a key substrate that underlies the emergence of spatially- and spectrally-patterned alterations in functional activity [23]. At its core, this model incorporates the effect of short- and long-range communication between cortical neuronal populations supported by the anatomical network. To test the proposed hypothesis, we performed a thorough parameter inference of the SGM on individual subjects’ source reconstructed MEG data and assessed parameters from a well-characterized clinical population of AD patients and a cohort of age-matched healthy controls. Consistent with our hypothesis, we demonstrate that a global slowing of long-range excitatory time scale is predictive of AD spatiospectral patterns and cognitive decline.

## 2 Methods

### 2.1 Data description

Eighty-eight patients with AD (diagnostic criteria for probable AD or mild cognitive impairment due to AD) [24–26] and 88 age-matched controls were included in this study. Each participant underwent a complete clinical history, physical examination, neuropsychological evaluation, brain magnetic resonance imaging (MRI), and a 5–10-minute session of resting MEG. All participants with AD were recruited from research cohorts at the University of California San Francisco-Alzheimer’s Disease Research Center(UCSF-ADRC). Healthy control participants were recruited at UCSF-ADRC as well as from several ongoing studies at the Biomagnetic Imaging Laboratory at UCSF. Informed consent was obtained from all participants and the study was approved by the Institutional Review Board (IRB) at UCSF (UCSF-IRB 10–02245). The mean (std) age of controls and patients with AD was 65.07 (9.92) and 62.73 (8.64) years, respectively. 51 (58%) of 88 controls, and 53 (60.2%) of patients with AD were females. The mean (std) MMSE score of patients with AD was 22.14 (5.55), while the mean Clinical Dementia Rating-Sum of Boxes (CDR) score of patients with AD was 4.90 (2.75).

### 2.2 Clinical assessments and MEG, and MRI acquisition and analyses

All the processing pipelines are the same as that for a previous study [20]. Patients with AD were assessed via MMSE and a standard battery of neuropsychological tests. Patients with AD were assessed via a structured caregiver interview to determine the Clinical Dementia Rating.

MEG scans were acquired on a whole-head biomagnetometer system (275 axial gradiometers; MISL, Co-quitlam, British Columbia, Canada) for 5–10 min, following the same protocols described previously [27, 20]. Tomographic reconstructions of source-space data were done using a continuous 60-second data epoch, an individualized head model based on structural MRI, and a frequency optimized adaptive spatial filtering technique implemented in the Neurodynamic Utility Toolbox for MEG (NUTMEG; http://nutmeg.berkeley.edu). We derived the regional power spectra based on Desikan–Killiany atlas parcellations for the 68 cortical regions depicting neocortex and allocortex, the latter including the entorhinal cortex. Regional power spectra were derived from FFT and then converted to dB scale.

### 2.3 Resting state MEG data acquisition

Each subject underwent MEG recording on a whole-head biomagnetometer system consisting of 275 axial gradiometers (MISL, Coquitlam, British Columbia, Canada), for 5–10 min. Three fiducial coils including nasion, left and right preauricular points were placed to localize the position of head relative to sensor array, and later coregistered to each individual’s respective MRI to generate an individualized head shape. Data collection was optimized to minimize within-session head movements and to keep it below 0.5 cm. 5–10 min of continuous recording was collected from each subject while lying supine and awake with eyes closed (sampling rate: 600 Hz). We selected a 60s (1 min) continuous segment with minimal artifacts (minimal excessive scatter at signal amplitude <10 pT), for each subject, for analysis. The study protocol required the participant to be interactive with the investigator and be awake at the beginning of the data collection. Spectral analysis of each MEG recording and whenever available, and the simultaneously collected scalp EEG recording were examined to confirm that the 60-s data epoch represented awake, eyes closed resting state for each participant. Artifact detection was confirmed by visual inspection of sensor data and channels with excessive noise within individual subjects were removed prior to analysis.

### 2.4 Source space reconstruction of MEG data and spectral power estimation

Tomographic reconstructions of the MEG data were generated using a head model based on each participant’s structural MRI. Spatiotemporal estimates of neural sources were generated using a time–frequency optimized adaptive spatial filtering technique implemented in the Neurodynamic Utility Toolbox for MEG (NUTMEG; https://nutmeg.berkeley.edu/). Tomographic volume of source locations (voxels) was computed through an adaptive spatial filter (10-mm lead field) that weights each location relative to the signal of the MEG sensors [28, 29]. The source space reconstruction approach provided amplitude estimations at each voxel derived through the linear combination of spatial weighting matrix with the sensor data matrix [28]. A high-resolution anatomical MRI was obtained for each subject (see below) and was spatially normalized to the Montreal Neurological Institute (MNI) template brain using the SPM software (http://www.fil.ion.ucl.ac.uk/spm), with the resulting parameters being applied to each individual subject’s source space reconstruction within the NUTMEG pipeline [29].

To prepare for source localization, all MEG sensor locations were coregistered to each subject’s anatomical MRI scans. The lead field (forward model) for each subject was calculated in NUTMEG using a multiple local-spheres head model (three-orientation lead field) and an 8-mm voxel grid which generated more than 5000 dipole sources, all sources were normalized to have a norm of 1. The MEG recordings were projected into source space using a beamformer spatial filter. Source estimates tend to have a bias towards superficial currents and the estimates are more error-prone when we approach subcortical regions, therefore, only the sources belonging to the 68 cortical regions were selected for further analyses. Specifically, all dipole sources were labeled based on the Desikan–Killiany parcellations, then sources within a 10-mm radial distance to the centroid of each brain region were extracted for each region. In this study, we examined the broad-band (1–35 Hz). Power spectra were derived by applying FFT on the time-course data and then converted to the dB scale.

### 2.5 Magnetic resonance image acquisition and analysis

Structural brain images were acquired from all participants using a unified MRI protocol on a 3 Tesla Siemens MRI scanner at the Neuroscience Imaging Center (NIC) at UCSF. Structural MRIs were used to generate individualized head models for source space reconstruction of MEG sensor data. Structural MRI scans were also used in the clinical evaluations of patients with AD to identify the pattern of gray matter volume loss to support the diagnosis of AD.

### 2.6 Model

SGM provides a closed-form solution of the steady-state frequency response of different brain regions. Here, we use a Desikan-Killiany parcellation scheme [30] to estimate the brain regions. The SGM is characterized by 7 parameters, which are either global or local but spatially-invariant. These parameters include the spatially-invariant local synchrony-related time constants $\tau_{e}$, $\tau_{i}$ and spatially-invariant but local neural gains $g_{ei}$, $g_{ii}$ as a measure of overall synaptic strength at the local scale for both excitatory and inhibitory neuronal subpopulations; as well as a global excitatory time constant $\tau_{G}$ at the long-range scale representing the long-range network connections, global coupling constant α, and speed of transmission of signals among regions v. Each region is assumed to consist of local excitatory and inhibitory neuronal subpopulations that interact with each other and regulate the long-range excitatory neuronal populations. The long-range populations are assumed to be connected to each other via the structural connectome. Here, we use a template structural connectome from the Human Connectome Project (HCP). Hence the model entails no features that may change from region to region, except of course features from the heterogeneously connected anatomical network. The structural connectivity matrix is shown in Supplementary Fig. S5 and the distance matrix is shown in Supplementary Fig. S6. To infer the SGM parameters, we fit SGM output to the frequency spectra obtained from MEG for healthy controls and AD subjects. The model used here is similar to the SGM developed previously [31–34], and is described in detail in the supplementary document.

The model solution can be obtained in a closed form in the frequency domain as a function of angular frequency ω as:

(1) $$X\left(\omega \right)=\sum_{k=1}^{N}\frac{u_{k}\left(\omega \right)u_{k}{\left(\omega \right)}^{\text{H}}}{\text{j}\omega \;+\;\tau_{\text{G}}^{-1}\lambda_{k}\left(\omega \right)F_{\text{G}}\left(\omega \right)}H_{\text{local}\;}\left(\omega \right)P\left(\omega \right),$$

where, X(ω) is the signal of every brain region, $u_{k}(\omega )$ are the eigenmodes and $\lambda_{k}(\omega )$ are the eigenvalues obtained by the eigen-decomposition of a complex Laplacian matrix. Equation (1) is the closed-form steady-state solution of the macroscopic signals at a specific angular frequency ω. We use this modeled spectra to compare against empirical MEG spectra and subsequently estimate model parameters. In practice, only a few eigenmodes k∈[1,K], K≪N are needed to obtain sufficiently strong fits to empirical data, including especially the lowest eigenmodes [32].

### 2.7 Model parameter estimation

The model parameter estimation procedure is same as described previously [34]. Modeled spectra was converted into PSD by calculating the norm of the frequency response and converting it to dB scale by taking $20{log}_{10}()$ of the norm. Pearson’s r between modeled PSD and the MEG PSD was used a goodness of fit metric for estimating model parameters. Pearson’s r between modeled and MEG PSD was computed for all 68 brain regions. Its average r across all regions is referred to as the *spectral correlation*. Next we calculated the *spatial correlation* by obtaining the regional distribution of alpha band (8–12 Hz) raw power of both model x and MEG y. Then, the spatial correlation was defined as $x^{T}∥(C+wI)∥y$, where C is the row degree normalized structural connectivity matrix, I is the identity matrix, w is an empirical weight, and ∥(C+10I)∥ is the row normalized version of C+10I. The objective function for optimization and estimation of model parameters was the sum of spectral and spatial correlations. We used a dual annealing optimization procedure in Python for performing parameter optimization [35].

Parameter initial guesses and bounds for estimating the static spectra are specified in Table 1. We defined three different bounds on the neural gain terms to ensure that the model is stable, based on prior work on model stability [33]. First, we supplied a larger bound on the neural gains for optimization. If the optimal model parameter was outside the stability boundary, we repeated optimization with a smaller bound. We repeated this procedure 3 times to ensure that the final optimal model parameters correspond to the stable model solutions. We used a dual annealing optimization procedure in Python for parameter optimization [35]. The dual annealing optimization was performed for three different initial guesses, and the parameter set leading to maximum sum of spectral and spatial correlations was chosen for each subject. The dual annealing settings were: maxiter = 500. All the other settings were the same as default.

### 2.8 Statistical analyses

Statistical tests were performed using SAS software (SAS9.4; SAS Institute, Cary, NC) and the stats-models package in Python. To compare the neuronal parameters between the controls and patients, we used a linear mixed-effects model (PROC MIXED), to compare model parameters $\left(\tau_{G},\tau_{e},\tau_{i},g_{ii},g_{ei},\alpha ,v\right)$, including age as a covariate into the models. We reported the estimated least-squares means and the statistical differences of least-squares means based on unpaired t-tests. We also developed univariate linear regression models to examine the associations between model parameters and MMSE and CDR scores in AD. In these models, the dependent variables included MMSE and CDR (in separate models), and the predictor variables included the model parameters we found significant between AD and controls ($\tau_{G},\;\tau_{e}$, and $g_{ii}$). Next, we developed multivariate linear regression models with dependent variables as MMSE and CDR (separately), and the predictor variables included all the significant parameters $\tau_{G},\;\tau_{e},\;g_{ii}$, and age as covariates.

### 2.9 Classification between AD and controls

We trained a random forest for classifying AD and controls. Here, we used the SGM parameters and age as features of the model. For training and testing, we employed a 5-fold stratified cross validation method. We divided the dataset into 5 folds and used the 4 folds for training, and the 5^th^ fold for testing the model. We repeated this procedure 100 times. While training the model, no information of the testing fold was provided. With the 4 folds of the training dataset, further 5-fold cross validation was performed to estimate the tuning parameter of the random forest. Here, we only tuned for the max depth with the following options for max depth: None, 2, 3, 4. All other hyperparameters were kept as default in the sklearn package in Python. After estimating the tuning parameter, the model was trained using the entire training dataset and then tested on the 5^th^ fold. The mean AUROC of the test dataset was finally reported. The feature importance was estimated as the average of the feature importance from the random forest classifier that was trained 100 times.

## 3 Results

### 3.1 Global timescales of empirical MEG electrophysiological recordings are longer in patients with AD

**Figure 1:**
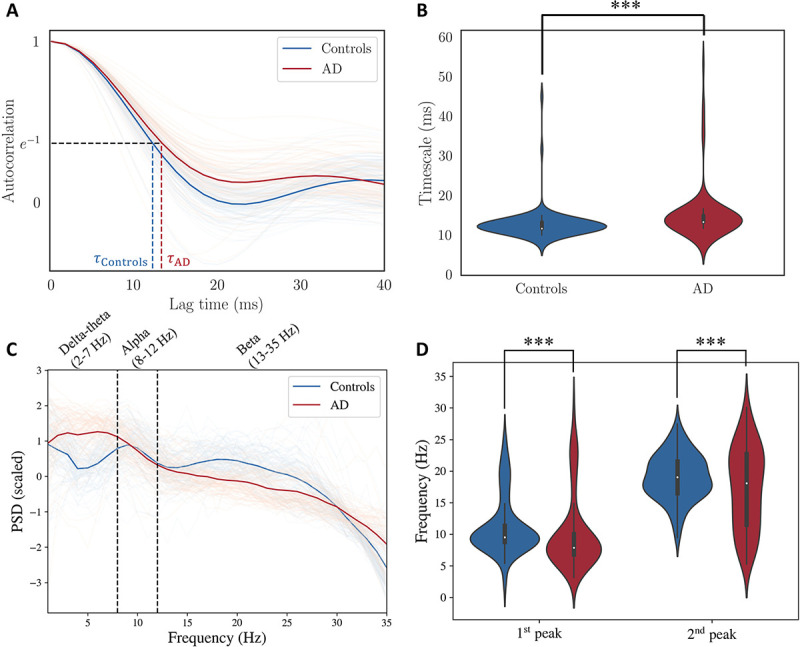
**A:** Autocorrelation function from MEG timeseries. Each autocorrelation function is a mean of autocorrelation functions of all the brain regions, and therefore each line corresponds to a single subject. The timescale is the value of lag that corresponds to an autocorrelation function value of $e^{-1}$. **B:** Distribution of timescales obtained from autocorrelation function. Based on a Kolmogorov-Smirnov test, time constant of MEG recordings for patients with AD is significantly larger than the timescale of MEG recordings for healthy controls (p<0.001, Cohen’s D effect size = 0.42). **C:** Power spectral density (PSD, in dB scale) extracted from the MEG recordings. Each PSD is a mean of all regions and then centered to the mean and scaled to unit variance for every subject separately. **D:** First and second peaks of the PSD in C for every subject. Both the first and the second peaks are lower in AD (p<0.001, Cohen’s D effect size = 0.21 for the first peak, p<0.001, Cohen’s D effect size = 0.32 for the second peak). The peaks were extracted using the FOOOF toolbox [37].

First, we show that the global timescales of empirical MEG recordings are longer in patients with AD. To this end, we collected MEG recordings for 88 patients with AD and 88 age-matched healthy controls. To evaluate the global timescales of the MEG recordings, we first calculated the autocorrelation function of the bandpass filtered MEG time-series for every brain region and then took a mean across all the brain regions (Fig. 1A). From this averaged autocorrelation function, we obtained an exponential decay time constant (τ in $e^{-t/\tau }$) that captures the timescale of a time series. Precisely, this is the value of the lag time for which the autocorrelation function is $e^{-1}$ (see [36] and the references therein for more details). As seen in Fig. 1B, the time constant is significantly longer for AD based on a Kolmogorov–Smirnov test, implying that the MEG signals of patients with AD decay slower. To supplement this result, we obtained the mean PSD over all regions (Fig. 1C) and compared the first and the second peaks of the PSD in C (Fig. 1D). As seen in Fig. 1D, both the first and the second peaks of the PSD are lower in AD, based on a Kolmogorov–Smirnov test. This decrease in the peak frequencies also indicates a slowing of the timescales in AD. To investigate the underlying mechanisms of this slowing, we inferred SGM parameters for the two cohorts as explained in the methods section and Supplementary Fig.S1.

### 3.2 SGM reliably reproduces the spectral and spatial patterns of power spectral density

**Figure 2:**
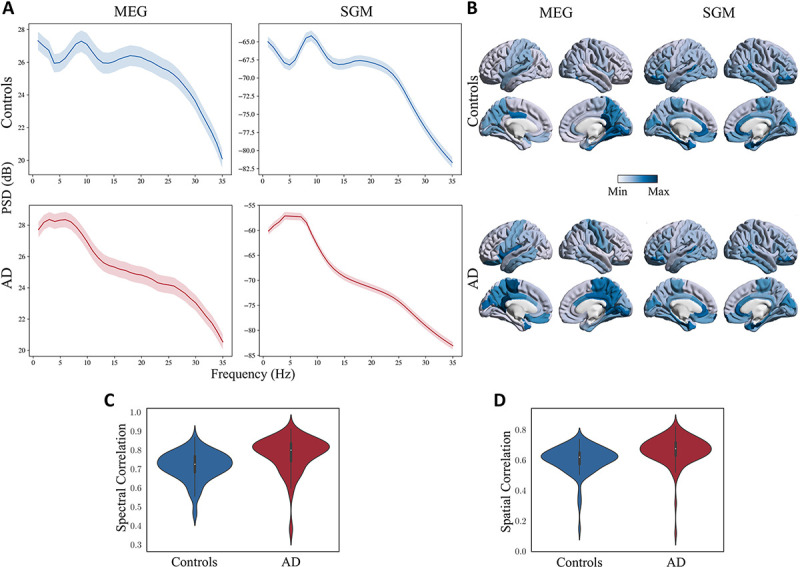
**A:** Comparison of empirical (left) and SGM (right) frequency spectra for controls (top) and patients with AD (bottom). The darker lines correspond to the PSD averaged over all regions and subjects. The shaded region corresponds to the 90% confidence interval for the mean PSD (over all subjects) for different regions. **B:** Spatial distribution of the empirical (left) and SGM (right) alpha frequency band, for subjects with mean spatial correlations in controls (top) and patients with AD (bottom). The color scale of each spatial distribution was chosen based on their dynamic range. **C:** Spectral correlations of model fitting for controls and patients with AD. **D:** Spatial correlations of model fitting for controls and patients with AD.

The predicted spectra from SGM reliably captured the empirical MEG spectra from patients with AD and age-matched controls (Fig. 2A; The mean (std) spectral correlations were 0.72 (0.08) and 0.78 (0.09) for controls and AD, respectively, shown in Fig. 2C). Compared to age-matched controls, patients with AD showed a reduced alpha peak and increased spectral power within the low-frequency delta-theta range (2–7 Hz), in their empirical spectral recording from MEG. This characteristic spectral change is clearly replicated in the predicted spectra derived from SGM. The spatial distribution of spectral power density of the alpha band, as expected, showed a postero-anterior distribution in both controls and patients. The spatial patterns of the predicted spectra from SGM reproduced this postero-anterior distribution with high fidelity (Fig. 2B; the mean (std) spatial correlations were 0.60 (0.09) and 0.66 (0.09) for controls and AD, respectively, shown in Fig. 2D). The region-wise spectral correlations and the frequency band-specific spatial correlations are also shown in Supplementary Fig.S2. These correlations were greater than 0.5 for more than 90% of subjects, regions, and frequency bands overall. Sample empirical versus modeled PSD for a single subject is shown in Supplementary Fig. S3. Sample spatial patterns in the beta frequency band are shown in Supplementary Fig. S4.

### 3.3 Patients with AD have altered network time constants and neural gains

**Figure 3:**
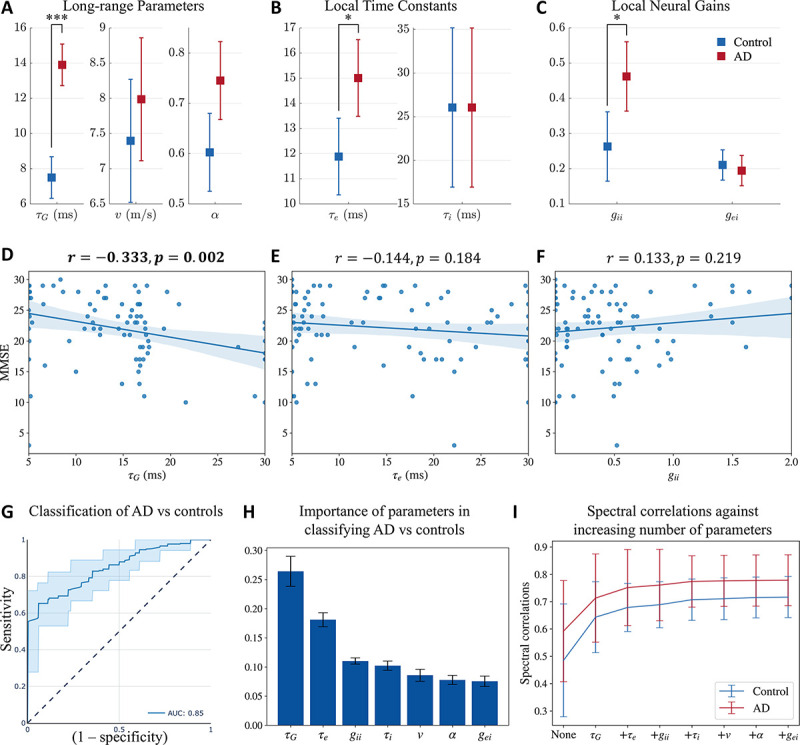
**A, B, C:** Statistical significance testing of difference in model parameters between AD and controls, with age as a covariate. Distribution of **A:** long-range parameters $\tau_{G}$ (long-range excitatory time constant), v (speed), and α (coupling constant); **B:** local time constants $\tau_{e}$ (excitatory) and $\tau_{i}$ (inhibitory); and **C:** local neural gains $g_{ii}$ (inhibitory gain) and $g_{ei}$ (gain of signals from the coupling between excitatory and inhibitory neurons). P-values are reported after correcting for multiple testing using a Bonferroni correction. ∗: *p* < 0.05, ∗ ∗ ∗: *p* < 0.001. **D, E, F:** Univariate associations of **D**: $\tau_{G}$, **E**: $\tau_{e}$, and **F**: $g_{ii}$ with MMSE in patients with AD. **G, H:** Classification of AD vs controls with a random forest classifier with SGM parameters and age as features of the classifier. **G:** ROC curve for classification of AD versus controls. **H:** Feature importance plot of SGM parameters. **I:** Spectral correlations when optimizing for only certain model parameters while keeping the others fixed at the average of the optimized model parameters of both AD and controls. “None” implies that all the model parameters were fixed at the average. The second point on the x-axis with the label $\tau_{G}$ implies that only $\tau_{G}$ was allowed to be optimized while the other model parameters were fixed at the average values. The third point on the x-axis with the label $\tau_{e}$ implies that both $\tau_{G}$ and $\tau_{e}$ were allowed to be optimized while keeping the other model parameters fixed at the average values. All the subsequent points on the x-axis correspond to similarly including more model parameters in optimization, based on their importance in the classification of AD vs controls.

Next, we compared the network parameters derived from the SGM between patients with AD and age-matched controls. Recall that these parameters are either global or local but assumed spatially-invariant. These parameters include the spatially-invariant local synchrony-related time constants $\tau_{e},\;\tau_{i}$ and spatially-invariant but local neural gains $g_{ei},\;g_{ii}$ as a measure of overall synaptic strength at the local scale for both excitatory and inhibitory neuronal subpopulations; as well as a global excitatory time constant $\tau_{G}$ at the long-range scale representing the long-range network connections, global coupling constant α, and speed of transmission of signals among regions v. Using a general linear model with age included as a covariate, we found that patients with AD have significantly elevated long-range excitatory time constant ($\tau_{G}$; controls mean = 7.50, confidence interval = (6.32, 8.68), AD mean = 13.90, confidence interval = (12.72, 15.09), Cohen’s D effect size = 1.16), local excitatory time constant, ($\tau_{e}$; controls mean = 11.88, confidence interval = (10.36, 13.41), AD mean = 15.01, confidence interval = (13.48, 16.53), Cohen’s D effect size = 0.41) and local inhibitory neural gain ($g_{ii}$; controls mean = 0.26, confidence interval = (0.16, 0.36), AD mean = 0.46, confidence interval = (0.36, 0.56), Cohen’s D effect size = 0.42; Fig. 3A, B, and C). The highest effect size among the parameter comparisons was found in $\tau_{G}$ between AD and controls. Collectively these results indicate that while global network parameters are altered in AD, long-range excitatory connections may reflect such changes with greater sensitivity than other parameters.

### 3.4 Altered long-range excitatory connections are correlated with global cognitive deficits in patients with AD

To investigate the association between altered global network parameters and cognitive deficits in patients with AD, we examined the correlations between $\tau_{G},\;\tau_{e}$, and $g_{ii}$ with global cognitive decline measured by Mini Mental State Exam (MMSE), and overall disease severity measured by clinical dementia rating sum of boxes (CDR), in patients with AD. We first tested for univariate associations between the model parameters and MMSE and CDR separately, using linear regression. After adjusting for multiple testing (Bonferroni), $\tau_{G}$ showed significant negative associations with MMSE (Fig. 3D) where higher $\tau_{G}$ predicted greater cognitive deficits in MMSE. Next, we tested for the association between $\tau_{G}$ and MMSE including $\tau_{e},\;g_{ii}$, and age as covariates in a multivariate linear regression model. This multivariate analysis also showed a significant negative association between $\tau_{G}$ and MMSE only (p=0.007 for the association between $\tau_{G}$ and MMSE, model r=0.402, model adjusted $r^{2}=0.121,\;F=3.961$). Similar to the univariate results, none of the parameters were significantly associated with CDR in a multivariate regression model after adjusting for multiple testing. Details of statistics are mentioned in the supplementary tables S1 and S2.

### 3.5 Altered global network parameters can distinguish between AD and controls with high accuracy

Next, we examined the sensitivity and specificity of altered global network parameters to distinguish between patients with AD and controls. To this end, we trained and tested a random forest classifier including the model parameters and age as the classifier features. The average AUC of the ROC curves from the testing folds is 0.85, with a standard deviation of 0.02 (Fig. 3G). The other classification metrics included: accuracy = 0.78, precision = 0.79, recall score = 0.75, and f1 score = 0.77, on average. The confusion matrix is shown in supplementary Fig. S7. We also obtained the feature importance score of the features used in training the model, shown in Fig. 3H. The time constant $\tau_{G}$ was the most important feature in classifying AD versus controls. Collectively, these results indicate that altered global network parameters are reliable indices to identify patients with AD from their age-matched counterparts and that long-range excitatory connections are the most sensitive indicators of AD-related global network deficits.

### 3.6 Minimal set of altered model parameters capture the empirical power spectral density patterns in AD

In order to assess the importance of model parameters in capturing the empirical PSD, we evaluated the spectral and spatial correlations after optimizing for certain model parameters, based on their importance from Fig. 3H, while keeping the remaining model parameters as the average of all the optimized model parameters for AD and controls together. First, we evaluated the correlations when none of the model parameters are optimized for and are all the average of the optimal parameters obtained previously. Next, we optimized only for $\tau_{G}$ while keeping all the other model parameters fixed since $\tau_{G}$ was the most important parameter in the classification of AD vs controls. Subsequently, we optimized for both $\tau_{G}$ and $\tau_{e}$ while keeping the remaining model parameters fixed since $\tau_{e}$ was the second most important feature in classification. We repeated this procedure till we included all the model parameters for optimization. The spectral correlations from this evaluation are reported in Fig. 3I. As seen in the figure, we see a sharp increase when $\tau_{G}$ is allowed to vary while keeping the other model parameters fixed. Upon including the subsequent model parameters, we do not see a substantial increase in the spectral correlation. This result strengthens our prior observation on the importance of $\tau_{G}$ in differentiating AD from controls. Note that we did not see any substantial difference in the spatial correlations.

## 4 Discussion

In this study, we determined local and long-range neuronal parameters of a computational model of brain activity that can account for abnormal neurophysiological activity in AD observed in high spatio-temporal resolution MEG imaging. We used SGM, which is ideally suited for this exploration providing a computational link between structure and function in the brain. The neuronal time constant associated with long-range excitatory connections is the most sensitive biophysical property that mediates abnormal global network dynamics in patients with AD. The long-range excitatory time constant not only predicts the global cognitive deficits in AD patients but also classifies AD versus controls with high accuracy. To our knowledge, this is the first report of a single global parameter change that can reliably reproduce spatial and spectral activity patterns in patients with AD and is also correlated with cognitive deficits. These findings provide critical insights about potential mechanistic links between abnormal neural oscillations and cellular correlates of impaired neuronal activity in AD.

### 4.1 Biophysical significance of significantly altered neuronal parameters

The parameters that were differentially distributed in AD were the excitatory time constants $\tau_{G}$ and $\tau_{e}$, and inhibitory neural gain $g_{ii}$. Each parameter has a distinct biophysical meaning, and clear implications in AD pathophysiology, as discussed below.

#### Long-range time constant.

The most important differential parameter was the long-range excitatory time constant $\tau_{G}$, which was (1) increased in AD; (2) capable of recapitulating the spectral shift seen in AD patients; (3) the most important feature in classifying AD from controls. Higher $\tau_{G}$ in AD indicates the slowing of long-range brain-wide communication of neural activity, implicating primarily the large layer-specific pyramidal glutamatergic neurons [38]. These pyramidal neurons are known to be selectively vulnerable in AD [39]. This result is also in concordance with a recent study demonstrating long-range axonal connectivity disruption in AD transgenic mice [40]. Increased pyramidal neuron time constants have also been reported in AD mouse models [41].

We also found that $\tau_{G}$ is also associated with global cognitive deficits in AD patients. This implicates impairment in the synaptic processing of long-range excitatory neurons, a potential factor contributing to increased $\tau_{G}$ in AD. Our recent study showed that alpha hyposynchrony is correlated with the degree of global cognitive dysfunction in patients with AD [27]. Another MEG-based study also demonstrated that oscillatory slowing predicts general as well as domain-specific cognitive function in patients with AD [21]. While such associations can be obtained using neuroimaging data directly, here we were able to identify a specific biophysically grounded parameter, $\tau_{G}$, that can potentially explain these biological relationships. Linking biophysical processes to clinical scales has historically been extremely challenging for conventional machine learning approaches due to the mismatch in dimensionality between input features (thousands) and output features (a handful of clinical measures). Increased long-range time constant in AD capable of recapitulating the spectral shifts in AD and correlated with MMSE, therefore, may be the first report of a single biophysical correlate accounting for clinical deficits in patients with AD.

#### Local excitatory time constant.

Interestingly, the local excitatory neural time constant was also significantly different in patients, whereas local inhibitory time constant was not. Local excitatory-inhibitory imbalances in AD have been demonstrated in numerous basic science studies [42, 20]. Our results are therefore broadly consistent with these studies, although with smaller effect sizes compared to the long-range time constant parameter. Among the local parameters, the strongest relationship was found with the local excitatory time constant $\tau_{e}$, which was higher in AD subjects than in controls, consistent with our previous findings. While higher $\tau_{e}$ implies the slowing of excitatory signals at the local level, we previously demonstrated that increased $\tau_{e}$ is distinctly associated with tau accumulations in AD [20]. The relationship between spatially invariant long-range excitatory time constant and regional tau accumulation in AD remains to be elucidated.

#### Local inhibitory neural gain

We also found that the inhibitory neural gain $g_{ii}$ is higher in AD subjects than in controls. Neural gains capture the overall synaptic strength. Higher $g_{ii}$ implies a higher synaptic strength of the inhibitory neuronal connections within a region. Alteration in the neural gain term indicates a neuronal excitatory-inhibitory imbalance; such an imbalance has been reported in various preclinical AD models [43–46]. Overall increased inhibitory gain was found in epilepsy [47], while the local subpopulation estimates in AD showed reductions as seen in our previous study [20]. This discrepancy requires further study in the future.

### 4.2 Global network effects versus local circuit effects

Two competing hypotheses can potentially account for the spatial and spectral abnormality patterns in AD: spatially variant effects of local circuits, and spatially invariant global network effects which is the focus of this study. Due to the highly specific spatial topography of AD pathology [48], prior literature has broadly focused on the neural correlates of local circuits as the primary means of describing observed electrophysiological data [42]. In a recent study, we tested the hypothesis that local changes in model parameters could recapitulate regional spectral shifts in AD patients. We also reported that alterations in local excitatory and inhibitory parameters are distinctly associated with tau and amyloid-β accumulations in AD patients [20]. The current study addresses a very different hypothesis: that observed spatiospectral changes in AD patients may be explained by *global* changes in the network, as compared to spatially-varying changes in local neural masses. While the two hypotheses on global network versus local circuit effects in AD are not mutually exclusive, our key contribution here is to show that global changes are sufficient to recapitulate the observed spatial and spectral abnormalities in AD. A previous modeling study also found differences in both coupling and local circuits [49]. Even though AD may induce both local and global changes, it is possible that the latter may dominate, as previously noted from a modeling perspective [50–54] and from our results indicating long-range $\tau_{G}$ as the most important parameter. Heterogeneity of the Amyloid-β load was previously found to be essential to simulate the slowing of rhythm [55], but we have demonstrated that spatial variations of any kind in our model are not needed to capture the spectral and alpha-band spatial patterns. This certainly leaves room for the possibility that the SGM will be enriched by including the spatial patterns of Amyloid-β and tau. Future explorations of the respective contributions of local versus global network changes in AD will be critical.

This local versus global distinction also means that our current results are not directly comparable to prior spatially-variable modeling results. Both the long-range ($\tau_{G}$) and the local ($\tau_{e}$ and $g_{ii}$) parameters in our SGM model showed significant group differences, but we did not reproduce other local changes reported, e.g., in [20]. Nevertheless, $\tau_{e}$ being higher in AD in both the local as well as global study indicate a common underlying mechanism involving excitatory neuronal subpopulations at both local and global level in AD.

### 4.3 Relationship to previous modeling works

Even though no mathematical model can capture the complex brain structure-function relationship completely, many can aid in identifying mechanisms that cannot be inferred with neuroimaging data alone. Indeed, various model-based markers of AD have also been shown in the past. For example, the Virtual Brain Modeling platform has been used to estimate local and global parameters of a neural mass model for fMRI and to subsequently differentiate between AD and controls [49]. While the literature on fMRI studies in AD is vast, comparable depth is lacking in the use of higher frequency data like MEG. In our work, we focus on MEG because it provides us with a high temporal resolution and can give insights into oscillatory signatures, especially the spectral and spatial patterns thereof, that are directly linked to cellular mechanisms. A neural mass modeling approach attributed slowing of alpha in AD using MEG to neuronal hyperactivity, though without directly fitting to the empirical data [56]. Another modeling approach examined different stimulation strategies to preserve functional network integrity in AD and found that stimulating excitatory neurons were the most successful [57]. Another virtual brain simulation approach integrated local field potential simulations with regional amyloid-β and tau uptake as empirical features to classify healthy controls, MCI, and AD and obtained an average F1 score of 0.743 [58] – our study reports a higher F1 score of 0.77 for classification of AD from controls with just a few parameters as features of a random forest classifier.

A key difference from prior modeling approaches is that our SGM is a linear model with a small set of biophysically interpretable global parameters. Therefore, it can be obtained in a closed-form solution in the frequency domain, and model parameter inference is more tractable. We employed SGM because prior studies indicate that the emergent macroscopic activity is independent of the microscopic activity of individual neurons [50–52, 59, 53, 54], and is primarily governed by the long-range connections [60–63]. Indeed, it was already demonstrated that SGM outperforms a Wilson-Cowan neural mass model in fitting the empirical MEG spectra [31]. A recent comparison showed that linear models outperformed non-linear models in predicting resting-state fMRI time series. This was attributed to the linearizing effects of macroscopic neurodynamics and neuroimaging due to spatial and temporal averaging, observation noise, and high dimensionality [64]. Given that the vast majority of computational models involving neural masses involve highly non-linear concepts like multistability, metastability, and other complex dynamics [65–69], it may be questioned whether AD-induced changes in brain macroscopic dynamics can even be reliably measured and robustly inferred. Instead, we expect that while neural activity in AD and health might be highly dynamic and non-linear, its macroscopic spatial and frequency patterns are known to be far more stable across individuals [51, 70, 71, 27, 20]. This is a key motivation for our use of the linear and deterministic SGM, which has demonstrable tractability and only a few free parameters capable of predicting spectral and regional profiles of MEG activity. To our knowledge, this is the first study identifying a parsimonious biophysically interpretable marker of AD and cognitive decline in AD.

### 4.4 Structural network harmonics are responsible for pathology transmission

It was previously shown by our group that the eigendecomposition of the graph Laplacian can be used to describe the spread of pathology as it ramifies within the brain’s anatomic connectivity network. It was demonstrated that only the eigenmodes corresponding to the lowest eigenvalues - named “persistent modes” are involved in AD pathology progression [72]. Since any aberration in local synchrony explored here must arise from the underlying progression of pathology in the AD brain, it is expected that the same or similar eigenmodes responsible for pathology progression may also be involved in aberrant synchrony. The SGM too can be decomposed into a small set of eigenmodes (see Eq 1). Remarkably, it was recently shown by our group that the lowest few eigenmodes of the SGM capture a large portion of the spatial distribution of alpha-band power [32], and are also important in explaining low-frequency long-range synchrony from fMRI [73]. This striking resemblance of eigenmodes of both pathological and electrophysiological processes establishes a conceptual bridge that has been hitherto unknown.

### 4.5 Alternative neuroimaging modalities for capturing synchrony

Resting-state functional MRI (fMRI), which measures the slow fluctuations of blood oxygenation signal in the brain as a proxy for neural activity [74], is also widely used to identify abnormal synchrony in AD. Leveraging graph-theoretic analyses, many studies now routinely describe the alterations in fMRI measures during the course of AD pathophysiology [48]. However, graph theoretic statistics of resting-state fMRI have shown inconsistent differences between patients with AD and healthy controls [75]. In addition, fMRI is limited in its ability to capture fast temporal scales of neuronal activity [76]. Electrophysiological techniques such as E/MEG address this limitation by capturing temporal activity scales with millisecond precision, though with a lower spatial resolution. Leveraging their temporal resolution, E/MEG can be used to infer the dynamic neural activity directly [77]. Given the higher spatial resolution of MEG as compared to EEG, we used MEG for quantifying synchrony in AD.

### 4.6 Limitations

To determine the shape of the power spectra we used Pearson’s R as the cost function. Future studies should aim at capturing the magnitude as well as selected spectral features. Here we employed the same template structural connectome from HCP for both cohorts, as it allowed us to pinpoint the biophysical alterations solely due to functional alterations. A prior study has also demonstrated that white matter network organization is preserved in AD [78]. However, it will be ideal to obtain individual structural connectomes in all individuals. In addition, we observed that the SGM fits better to spectral and spatial patterns from AD rather than from controls. This may be attributed to the spectral shape of AD – it has a clearer exponential fall-off that is easier to fit to. In comparison, the spectral shape of controls has an additional peak in the beta band superimposed on the exponential fall-off. Lastly, we note that even though no mathematical model can capture the complex brain structure-function relationship completely, many can aid in identifying mechanisms that cannot be inferred with neuroimaging data alone.

### 4.7 Conclusion

This work shows that a global impairment in the excitatory long-range pyramidal neuronal population is the most important indicator of AD, and is also associated with global cognitive decline in patients with AD. Intriguingly, our work is able to recapitulate the spatial and spectral patterns of AD-related functional activity without introducing any spatial heterogeneity; indeed, the SGM model is entirely global and spatially-invariant. This raises the possibility that a global increase in the long-range excitatory time constant might be a sufficient factor underlying observed spatiotemporal alterations of neuronal activity in AD. This modeling approach highlights a parsimonious framework for identifying cellular biomarkers of abnormal electrophysiological oscillations and cognitive deficits in AD, that can aid in guiding future clinical trials.

## Supplementary Material

Supplement 1

## Figures and Tables

**Table 1:** SGM parameter values, initial guesses, and bounds for parameter estimation for static spectra fitting

Name	Symbol	Initial value 1	Initial value 2	Initial value 3	Lower/upper bound for optimization
Excitatory time constant	*τ*_e_	0.015 s	0.025 s	0.006 s	[0.005 s, 0.03 s]
Inhibitory time constant	*τ*_i_	0.01 s	0.08 s	0.15 s	[0.005 s, 0.2 s]
Long-range connectivity coupling constant	*α*	1	0.5	0.1	[0.1, 1]
Transmission speed	*υ*	5 m/s	10 m/s	18 m/s	[5 m/s, 20 m/s]
Alternating population gain	*g*_ei_	0.3	0.2	0.1	[0.001,0.7], [0.001,0.5], [0.001,0.4]
Inhibitory gain	*g*_ii_	0.6	0.1	1.2	[0.001,2.0], [0.001,1.5], [0.001,1.5]
Graph time constant	*τ*_G_	0.006 s	0.015 s	0.025 s	[0.005 s, 0.03 s]
Excitatory gain	*g*_ee_	n/a	n/a	n/a	n/a

## Data Availability

The code for this work is available here: https://github.com/parulv1/sgm-ad. Anonymized subject data will be shared on request from qualified investigators for the purposes of replicating procedures and results, and for other non-commercial research purposes within the limits of participants’ consent. Material requests should be addressed to Kamalini.ranasinghe@ucsf.edu.

## References

[1] Uhlhaas Peter J and Singer Wolf. Neural synchrony in brain disorders: relevance for cognitive dysfunctions and pathophysiology. neuron, 52(1):155–168, 2006. 17015233 10.1016/j.neuron.2006.09.020

[2] Wang Ruofan, Wang Jiang, Yu Haitao, Wei Xile, Yang Chen, and Deng Bin. Power spectral density and coherence analysis of Alzheimer’s EEG. Cognitive Neurodynamics, 9(3):291–304, 2015. 25972978 10.1007/s11571-014-9325-x PMC4427585

[3] Sedghizadeh Mohammad, Aghajan Hamid, Zahra Vahabi, Fatemi Seyyedeh, and Afzal Arshia. Network synchronization deficits caused by dementia and Alzheimer’s disease serve as topographical biomarkers: a pilot study. Brain Structure and Function, 227:2957—−2969, 2022. 35997832 10.1007/s00429-022-02554-2 PMC9396580

[4] Sperling Reisa A., LaViolette Peter S, O’Keefe Kelly, O’Brien Jacqueline, Rentz Dorene M, Pihlajamaki Maija, Marshall Gad, Hyman Bradley T., Selkoe Dennis J., Hedden Trey, Buckner Randy L., Alex Becker J., and Johnson Keith A. Amyloid Deposition Is Associated with Impaired Default Network Function in Older Persons without Dementia. Neuron, 63(2):178–188, 2009. 19640477 10.1016/j.neuron.2009.07.003 PMC2738994

[5] Jones David T., Knopman David S., Gunter Jeffrey L., Jonathan Graff-Radford Prashanthi Vemuri, Boeve Bradley F., Petersen Ronald C., Cascading network failure across the Alzheimer’s disease spectrum. Brain, 139(2):547–562, 11 2015. 26586695 10.1093/brain/awv338 PMC4805086

[6] Engels M.M.A., van der Flier W.M., Stam C.J., Hillebrand A., Scheltens Ph, and van Straaten E.C.W. Alzheimer’s disease: The state of the art in resting-state magnetoencephalography. Clinical Neurophysiology, 128(8):1426–1437, 2017. 28622527 10.1016/j.clinph.2017.05.012

[7] Mandal Pravat K., Banerjee Anwesha, Tripathi Manjari, and Sharma Ankita. A Comprehensive Review of Magnetoencephalography (MEG) Studies for Brain Functionality in Healthy Aging and Alzheimer’s Disease (AD). Frontiers in Computational Neuroscience, 12, 2018. 10.3389/fncom.2018.00060 PMC6115612 30190674

[8] M Penttilä J. Partanen V, Soininen H, and Riekkinen P.J. Quantitative analysis of occipital eeg in different stages of alzheimer’s disease. Electroencephalography and Clinical Neurophysiology, 60(1):1–6, 1985. 2578347 10.1016/0013-4694(85)90942-3

[9] Ursula Schreiter-Gasser Theo Gasser, and Ziegler Peter. Quantitative eeg analysis in early onset alzheimer’s disease: a controlled study. Electroencephalography and Clinical Neurophysiology, 86(1):15–22, 1993. 7678387 10.1016/0013-4694(93)90063-2

[10] C Huang L.-Wahlund O, Dierks T, Julin P, Winblad B, and Jelic V. Discrimination of alzheimer’s disease and mild cognitive impairment by equivalent eeg sources: a cross-sectional and longitudinal study. Clinical Neurophysiology, 111(11):1961–1967, 2000. 11068230 10.1016/s1388-2457(00)00454-5

[11] Dauwels Justin, Vialatte Francois, and Cichocki Andrzej. Diagnosis of alzheimer’s disease from eeg signals: where are we standing? Current Alzheimer Research, 7(6):487–505, 2010. 20455865 10.2174/156720510792231720

[12] Justin Dauwels K M Ramasubba Reddy Srinivasan, Musha Toshimitsu, Vialatte François-Benoît, Latchoumane Charles, Jeong Jaeseung, Cichocki Andrzej, Slowing and loss of complexity in alzheimer’s eeg: two sides of the same coin? International journal of Alzheimer’s disease, 2011, 2011. 10.4061/2011/539621 PMC3090755 21584257

[13] Jafari Zahra, Kolb Bryan E., and Mohajerani Majid H.. Neural oscillations and brain stimulation in alzheimer’s disease. Progress in Neurobiology, 194:101878, 2020. 32615147 10.1016/j.pneurobio.2020.101878

[14] W Berendse H., A Verbunt J.P., Ph Scheltens B. van Dijk W, and Jonkman E.J. Magnetoencephalographic analysis of cortical activity in alzheimer’s disease: a pilot study. Clinical Neurophysiology, 111(4):604–612, 2000. 10727911 10.1016/s1388-2457(99)00309-0

[15] Alberto Fernández Fernando Maestú, Amo Carlos, Gil Pedro, Fehr Thorsten, Wienbruch Christian, Rockstroh Brigitte, Elbert Thomas, and Ortiz Tomás. Focal temporoparietal slow activity in alzheimer’s disease revealed by magnetoencephalography. Biological Psychiatry, 52(7):764–770, 2002. 12372668 10.1016/s0006-3223(02)01366-5

[16] Osipova Daria, Ahveninen Jyrki, Jensen Ole, Ylikoski Ari, and Pekkonen Eero. Altered generation of spontaneous oscillations in alzheimer’s disease. NeuroImage, 27(4):835–841, 2005. 15961323 10.1016/j.neuroimage.2005.05.011

[17] Fernandez Alberto, Hornero Roberto, Mayo Agustın, Poza Jesus, Gil-Gregorio Pedro, and Ortiz Tomas. MEG spectral profile in Alzheimer’s disease and mild cognitive impairment. Clinical Neurophysiology, 117(2):306–314, 2006. 16386951 10.1016/j.clinph.2005.10.017

[18] de Haan Willem, Stam Cornelis J, Jones Bethany F, Zuiderwijk Ilonka M, van Dijk Bob W, and Scheltens Philip. Resting-state oscillatory brain dynamics in alzheimer disease. Journal of Clinical Neurophysiology, 25(4):187–193, 2008. 18677182 10.1097/WNP.0b013e31817da184

[19] David López-Sanz Noelia Serrano, and Fernando Maestú. The role of magnetoencephalography in the early stages of alzheimer’s disease. Frontiers in Neuroscience, 12, 2018. 10.3389/fnins.2018.00572 PMC6104188 30158852

[20] Ranasinghe Kamalini, Verma Parul, Cai Chang, Xie Xihe, Kudo Kiwamu, Gao Xiao, Lerner Hannah, Mizuiri Danielle, Strom Amelia, Iaccarino Leonardo, Renaud La Joie, Bruce L Miller, Maria Luisa Gorno-Tempini, Katherine P Rankin, William J Jagust, Keith Vossel, Gil Rabinovici, Ashish Raj, and Srikantan Nagarajan. Altered excitatory and inhibitory neuronal subpopulation parameters are distinctly associated with tau and amyloid in alzheimer’s disease. eLife, 11:e77850, may 2022. 35616532 10.7554/eLife.77850 PMC9217132

[21] Wiesman Alex I, Murman Daniel L, Losh Rebecca A, Schantell Mikki, Christopher-Hayes Nicholas J, Johnson Hallie J, Willett Madelyn P, Wolfson Sara L, Losh Kathryn L, Johnson Craig M, May Pamela E, and Wilson Tony W. Spatially resolved neural slowing predicts impairment and amyloid burden in Alzheimer’s disease. Brain, 145(6):2177–2189, 01 2022. 35088842 10.1093/brain/awab430 PMC9246709

[22] Nakamura Akinori, Cuesta Pablo, Alberto Fernández Yutaka Arahata, Iwata Kaori, Kuratsubo Izumi, Bundo Masahiko, Hattori Hideyuki, Sakurai Takashi, Electromagnetic signatures of the preclinical and prodromal stages of Alzheimer’s disease. Brain, 141(5):1470–1485, 03 2018. 29522156 10.1093/brain/awy044 PMC5920328

[23] Buzsaki Gyorgy. Rhythms of the Brain. Oxford university press, 2006.

[24] Albert Marilyn S., DeKosky Steven T., Dickson Dennis, Dubois Bruno, Feldman Howard H, Fox Nick C, Gamst Anthony, Holtzman David M, . The diagnosis of mild cognitive impairment due to Alzheimer’s disease: Recommendations from the National Institute on Aging-Alzheimer’s Association workgroups on diagnostic guidelines for Alzheimer’s disease. Alzheimer’s & Dementia, 7(3):270–279, 2011. 10.1016/j.jalz.2011.03.008 PMC3312027 21514249

[25] McKhann Guy M., Knopman David S, Chertkow Howard, Hyman Bradley T, Jack Clifford R Jr., Kawas Claudia H, Klunk William E, . The diagnosis of dementia due to Alzheimer’s disease: Recommendations from the National Institute on Aging-Alzheimer’s Association workgroups on diagnostic guidelines for Alzheimer’s disease. Alzheimer’s & Dementia, 7(3):263–269, 2011. 10.1016/j.jalz.2011.03.005 PMC3312024 21514250

[26] Jack Clifford R. Jr., Bennett David A., Blennow Kaj, Carrillo Maria C., Dunn Billy, Samantha Budd Haeberlein David M. Holtzman, Jagust William, NIA-AA Research Framework: Toward a biological definition of Alzheimer’s disease. Alzheimer’s & Dementia, 14(4):535–562, 2018. 10.1016/j.jalz.2018.02.018 PMC5958625 29653606

[27] Kamalini G Ranasinghe Jungho Cha, Iaccarino Leonardo, Hinkley Leighton B, Beagle Alexander J, Pham Julie, Jagust William J, Miller Bruce L, Rankin Katherine P, Rabinovici Gil D, Neurophysiological signatures in Alzheimer’s disease are distinctly associated with TAU, amyloid-β accumulation, and cognitive decline. Science Translational Medicine, 12(534):eaaz4069, 2020. 32161102 10.1126/scitranslmed.aaz4069 PMC7138514

[28] Dalal Sarang S., Guggisberg Adrian G., Edwards Erik, Sekihara Kensuke, Findlay Anne M., Canolty Ryan T., Berger Mitchel S., Knight Robert T., Barbaro Nicholas M., Kirsch Heidi E., and Nagarajan Srikantan S.. Five-dimensional neuroimaging: Localization of the time–frequency dynamics of cortical activity. NeuroImage, 40(4):1686–1700, 2008. 18356081 10.1016/j.neuroimage.2008.01.023 PMC2426929

[29] Dalal Sarang S, Zumer Johanna M, Guggisberg Adrian G, Trumpis Michael, Wong Daniel DE, Sekihara Kensuke, and Nagarajan Srikantan S. MEG/EEG source reconstruction, statistical evaluation, and visualization with NUTMEG. Computational intelligence and neuroscience, 2011, 2011. 10.1155/2011/758973 PMC3061455 21437174

[30] Rahul S Desikan Florent Ségonne, Fischl Bruce, Quinn Brian T, Dickerson Bradford C, Blacker Deborah, Buckner Randy L, Dale Anders M, Maguire R Paul, Hyman Bradley T, An automated labeling system for subdividing the human cerebral cortex on MRI scans into gyral based regions of interest. Neuroimage, 31(3):968–980, 2006. 16530430 10.1016/j.neuroimage.2006.01.021

[31] Raj Ashish, Cai Chang, Xie Xihe, Palacios Eva, Owen Julia, Mukherjee Pratik, and Nagarajan Srikantan. Spectral graph theory of brain oscillations. Human Brain Mapping, 41(11):2980–2998, 2020. 32202027 10.1002/hbm.24991 PMC7336150

[32] Verma Parul, Nagarajan Srikantan, and Raj Ashish. Spectral graph theory of brain oscillations—-revisited and improved. NeuroImage, 249:118919, 2022. 35051584 10.1016/j.neuroimage.2022.118919 PMC9506601

[33] Verma Parul, Nagarajan Srikantan, and Raj Ashish. Stability and dynamics of a spectral graph model of brain oscillations. Network Neuroscience, pages 1–43, 07 2022. 37334000 10.1162/netn_a_00263 PMC10270709

[34] Raj Ashish, Verma Parul, and Nagarajan Srikantan. Structure-function models of temporal, spatial, and spectral characteristics of non-invasive whole brain functional imaging. Frontiers in neuroscience, 16:959557–959557, 2022. 36110093 10.3389/fnins.2022.959557 PMC9468900

[35] Y Xiang D. Y Sun, W Fan, and X.G Gong. Generalized simulated annealing algorithm and its application to the Thomson model. Physics Letters A, 233(3):216–220, 1997.

[36] Gao Richard, van den Brink Ruud L, Pfeffer Thomas, and Voytek Bradley. Neuronal timescales are functionally dynamic and shaped by cortical microarchitecture. eLife, 9:e61277, nov 2020. 33226336 10.7554/eLife.61277 PMC7755395

[37] Donoghue Thomas, Haller Matar, Erik J Peterson Paroma Varma, Sebastian Priyadarshini, Gao Richard, Noto Torben, Lara Antonio H, Wallis Joni D, Knight Robert T, Parameterizing neural power spectra into periodic and aperiodic components. Nature neuroscience, 23(12):1655–1665, 2020. 33230329 10.1038/s41593-020-00744-x PMC8106550

[38] Brown Solange Pand Hestrin Shaul. Intracortical circuits of pyramidal neurons reflect their long-range axonal targets. Nature, 457(7233):1133–1136, 2009. 19151698 10.1038/nature07658 PMC2727746

[39] Luebke Jennifer I, Weaver Christina M, Rocher Anne B, Rodriguez Alfredo, Crimins Johanna L, Dickstein Dara L, Wearne Susan L, and Hof Patrick R. Dendritic vulnerability in neurodegenerative disease: insights from analyses of cortical pyramidal neurons in transgenic mouse models. Brain Structure and Function, 214(2):181–199, 2010. 20177698 10.1007/s00429-010-0244-2 PMC3045830

[40] Yuan Peng, Zhang Mengyang, Tong Lei, Thomas M Morse, Robert A McDougal, Hui Ding, Diane Chan, Yifei Cai, and Jaime Grutzendler. PLD3 affects axonal spheroids and network defects in Alzheimer’s disease. Nature, pages 1–10, 2022. 10.1038/s41586-022-05491-6 PMC9729106 36450991

[41] Islam Anam, Saito Takashi, Saido Takaomi, and Ali Afia B.. Presubiculum principal cells are preserved from degeneration in knock-in APP/TAU mouse models of Alzheimer’s disease. Seminars in Cell Developmental Biology, 139:55–72, 2023. Special Issue: Alzheimer’s Disease: Effects on Brain Circuits and Synapses. 35292192 10.1016/j.semcdb.2022.03.001 PMC10439011

[42] Eva Vico Varela Guillaume Etter, and Williams Sylvain. Excitatory-inhibitory imbalance in Alzheimer’s disease and therapeutic significance. Neurobiology of Disease, 127:605–615, 2019. 30999010 10.1016/j.nbd.2019.04.010

[43] Jorge J Palop A network dysfunction perspective on neurodegenerative diseases. Nature, 443(7113):768–773, 2006. 17051202 10.1038/nature05289

[44] Fernando Maestú Pablo Cuesta, Hasan Omar, Alberto Fernandéz Michael Funke, and Schulz Paul E.. The Importance of the Validation of M/EEG With Current Biomarkers in Alzheimer’s Disease. Frontiers in Human Neuroscience, 13, 2019. 10.3389/fnhum.2019.00017 PMC6374629 30792632

[45] Harris Samuel S. Tipping the Scales: Peptide-Dependent Dysregulation of Neural Circuit Dynamics in Alzheimer’s Disease. Neuron, 107(3):417–435, 2020. 32579881 10.1016/j.neuron.2020.06.005

[46] Chang Siyuan, Wang Jiang, Liu Chen, Yi Guosheng, Lu Meili, Che Yanqiu, and Wei Xile. A data driven experimental system for individualized brain stimulation design and validation. IEEE Transactions on Neural Systems and Rehabilitation Engineering, 29:1848–1857, 2021. 34478377 10.1109/TNSRE.2021.3110275

[47] Elahian Bahareh, Lado Nathan E., Mankin Emily, Vangala Sitaram, Misra Amrit, Moxon Karen, Fried Itzhak, Sharan Ashwini, Yeasin Mohammed, Staba Richard, Bragin Anatol, Avoli Massimo, Sperling Michael R., Jerome Engel Jr, and Shennan A. Weiss. Low-voltage fast seizures in humans begin with increased interneuron firing. Annals of Neurology, 84(4):588–600, 2018. 30179277 10.1002/ana.25325 PMC6814155

[48] Jagust William. Imaging the evolution and pathophysiology of Alzheimer disease. Nature Reviews Neuroscience, 19(11):687–700, 2018. 30266970 10.1038/s41583-018-0067-3 PMC7032048

[49] Zimmermann J., Perry A., Breakspear M., Schirner M., Sachdev P., Wen W., Kochan N. A., Mapstone M., Ritter P., McIntosh A. R., and Solodkin A.. Differentiation of Alzheimer’s disease based on local and global parameters in personalized Virtual Brain models. NeuroImage: Clinical, 19:240–251, 2018. 30035018 10.1016/j.nicl.2018.04.017 PMC6051478

[50] Shimizu H. and Haken H.. Co-operative dynamics in organelles. Journal of Theoretical Biology, 104(2):261–273, 1983. 6227776 10.1016/0022-5193(83)90414-9

[51] Peter A Robinson CJ Rennie, Donald L Rowe, SC O’Connor, and E Gordon. Multiscale brain modelling. Philosophical Transactions of the Royal Society B: Biological Sciences, 360(1457):1043–1050, 2005. 10.1098/rstb.2005.1638 PMC1854922 16087447

[52] Destexhe Alain and Sejnowski Terrence J. The Wilson–Cowan model, 36 years later. Biological cybernetics, 101(1):1–2, 2009. 19662434 10.1007/s00422-009-0328-3 PMC2866289

[53] Bratislav Mišic Olaf Sporns, and Anthony R McIntosh. Communication efficiency and congestion of´ signal traffic in large-scale brain networks. PLoS Comput Biol, 10(1):e1003427, 2014. 24415931 10.1371/journal.pcbi.1003427 PMC3886893

[54] Mišic Bratislav, Betzel Richard F, Nematzadeh Azadeh, o Joaquin, Griffa Alessandra, Hagmann Patric, Flammini Alessandro, Ahn Yong-Yeol, and Sporns Olaf. Cooperative and competitive spreading dynamics on the human connectome. Neuron, 86(6):1518–1529, 2015. 26087168 10.1016/j.neuron.2015.05.035

[55] Stefanovski Leon, Triebkorn Paul, Spiegler Andreas, Margarita-Arimatea Diaz-Cortes Ana Solodkin, Jirsa Viktor, McIntosh Anthony Randal, Linking molecular pathways and large-scale computational modeling to assess candidate disease mechanisms and pharmacodynamics in Alzheimer’s disease. Frontiers in computational neuroscience, page 54, 2019. 31456676 10.3389/fncom.2019.00054 PMC6700386

[56] van Nifterick Anne M, Gouw, van Kesteren Ronald E, h Philip, Stam Cornelis J, and de Haan Willem. A multiscale brain network model links Alzheimer’s disease-mediated neuronal hyperactivity to large-scale oscillatory slowing. Alzheimer’s research & therapy, 14(1):1–20, 2022. 10.1186/s13195-022-01041-4 PMC9310500 35879779

[57] de Haan Willem, van Straaten Elisabeth C. W, Gouw Alida A, and Stam Cornelis J. Altering neuronal excitability to preserve network connectivity in a computational model of Alzheimer’s disease. PLOS Computational Biology, 13(9):1–23, 09 2017. 10.1371/journal.pcbi.1005707 PMC5627940 28938009

[58] Triebkorn Paul, Stefanovski Leon, Dhindsa Kiret, Margarita-Arimatea Diaz-Cortes Patrik Bey, Konstantin Bülau Roopa Pai, Spiegler Andreas, Solodkin Ana, Jirsa Viktor, Brain simulation augments machine-learning–based classification of dementia. Alzheimer’s & Dementia: Translational Research & Clinical Interventions, 8(1):e12303, 2022. 35601598 10.1002/trc2.12303 PMC9107774

[59] Abdelnour Farras, Voss Henning U., and Raj Ashish. Network diffusion accurately models the relationship between structural and functional brain connectivity networks. NeuroImage, 90:335–347, 2014. 24384152 10.1016/j.neuroimage.2013.12.039 PMC3951650

[60] Jirsa V.K., Jantzen K.J., Fuchs A., and Kelso J.A.S.. Spatiotemporal forward solution of the eeg and meg using network modeling. IEEE Transactions on Medical Imaging, 21(5):493–504, 2002. 12071620 10.1109/TMI.2002.1009385

[61] Deco Gustavo, Senden Mario, and Jirsa Viktor. How anatomy shapes dynamics: a semi-analytical study of the brain at rest by a simple spin model. Frontiers in Computational Neuroscience, 6:68, 2012. 23024632 10.3389/fncom.2012.00068 PMC3447303

[62] Nakagawa Tristan T., Woolrich Mark, Luckhoo Henry, Joensson Morten, Mohseni Hamid, Kringelbach Morten L., Jirsa Viktor, and Deco Gustavo. How delays matter in an oscillatory whole-brain spiking-neuron network model for MEG alpha-rhythms at rest. NeuroImage, 87:383–394, 2014. 24246492 10.1016/j.neuroimage.2013.11.009

[63] F Abdelnour A Raj M Dayan, Devinsky O, and Thesen T. Estimating function from structure in epileptics using graph diffusion model. In 2015 IEEE 12th International Symposium on Biomedical Imaging (ISBI), pages 466–469, 2015.

[64] Nozari Erfan, Stiso Jennifer, Caciagli Lorenzo, Cornblath Eli J., He Xiaosong, Bertolero Maxwell A., Mahadevan Arun S., Pappas George J., and Bassett Danielle S.. Is the brain macroscopically linear? a system identification of resting state dynamics. bioRxiv, 2020.

[65] Freyer Frank, Roberts James A., Becker Robert, Robinson Peter A., Ritter Petra, and Breakspear Michael. Biophysical Mechanisms of Multistability in Resting-State Cortical Rhythms. Journal of Neuroscience, 31(17):6353–6361, 2011. 21525275 10.1523/JNEUROSCI.6693-10.2011 PMC6622680

[66] Deco Gustavo and Jirsa Viktor K.. Ongoing Cortical Activity at Rest: Criticality, Multistability, and Ghost Attractors. Journal of Neuroscience, 32(10):3366–3375, 2012. 22399758 10.1523/JNEUROSCI.2523-11.2012 PMC6621046

[67] Cabral Joana, Kringelbach Morten L., and Deco Gustavo. Exploring the network dynamics underlying brain activity during rest. Progress in Neurobiology, 114:102–131, 2014. 24389385 10.1016/j.pneurobio.2013.12.005

[68] Golos Mathieu, Jirsa Viktor, and Daucé Emmanuel. Multistability in Large Scale Models of Brain Activity. PLOS Computational Biology, 11(12):1–32, 12 2016. 10.1371/journal.pcbi.1004644 PMC4692486 26709852

[69] Deco Gustavo, Kringelbach Morten L, Jirsa Viktor K, and Ritter Petra. The dynamics of resting fluctuations in the brain: metastability and its dynamical cortical core. Scientific reports, 7(1):1–14, 2017. 28596608 10.1038/s41598-017-03073-5 PMC5465179

[70] Freeman Walter Jand Zhai Jian. Simulated power spectral density (PSD) of background electrocorticogram (ECoG). Cognitive neurodynamics, 3(1):97–103, 2009. 19003455 10.1007/s11571-008-9064-y PMC2645494

[71] He Biyu J., Zempel John M., Snyder Abraham Z., and Raichle Marcus E.. The Temporal Structures and Functional Significance of Scale-free Brain Activity. Neuron, 66(3):353–369, 2010. 20471349 10.1016/j.neuron.2010.04.020 PMC2878725

[72] Raj Ashish, Kuceyeski Amy, and Weiner Michael. A network diffusion model of disease progression in dementia. Neuron, 73(6):1204–1215, 2012. 22445347 10.1016/j.neuron.2011.12.040 PMC3623298

[73] Xie Chang Cai, Damasceno Pablo F., Nagarajan Srikantan S., and Raj Ashish. Emergence of canonical functional networks from the structural connectome. NeuroImage, 237:118190, 2021. 34022382 10.1016/j.neuroimage.2021.118190 PMC8451304

[74] Ogawa Seiji, Lee Tso-Ming, Kay Alan R, and Tank David W. Brain magnetic resonance imaging with contrast dependent on blood oxygenation. proceedings of the National Academy of Sciences, 87(24):9868–9872, 1990. 10.1073/pnas.87.24.9868 PMC55275 2124706

[75] Bachmann Claudia, Heidi IL Jacobs PierGianLuca Porta Mana, Dillen Kim, Richter Nils, Boris Von Reutern Julian Dronse, Oezguer A Onur Karl-Josef Langen, Fink Gereon R, On the extraction and analysis of graphs from resting-state fmri to support a correct and robust diagnostic tool for alzheimer’s disease. Frontiers in neuroscience, 12:528, 2018. 30323734 10.3389/fnins.2018.00528 PMC6172342

[76] Parker Andrew, Derrington Andrew, Blakemore Colin, and Logothetis Nikos K.. The neural basis of the blood–oxygen–level–dependent functional magnetic resonance imaging signal. Philosophical Transactions of the Royal Society of London. Series B: Biological Sciences, 357(1424):1003–1037, 2002. 12217171 10.1098/rstb.2002.1114 PMC1693017

[77] Baillet Sylvain. Magnetoencephalography for brain electrophysiology and imaging. Nature neuroscience, 20(3):327–339, 2017. 28230841 10.1038/nn.4504

[78] Powell Fon, Tosun Duygu, Sadeghi Roksana, Weiner Michael, Raj Ashish, Alzheimer’s Disease Neuroimaging Initiative, et al. Preserved structural network organization mediates pathology spread in Alzheimer’s disease spectrum despite loss of white matter tract integrity. Journal of Alzheimer’s Disease, 65(3):747–764, 2018. 10.3233/JAD-170798 PMC6152926 29578480

