# Supplementary material for: Impaired long-range excitatory time scale predicts abnormal neural oscillations and cognitive deficits in Alzheimer’s disease
Source: Res Sq. 2023 Nov 1:rs.3.rs-2579392. Originally published 2023 Mar 22. Preprint. [Version 3] doi: 10.21203/rs.3.rs-2579392/v3 (PMC10055509; doi:10.21203/rs.3.rs-2579392/v3)
Supplement: Supplement 1 [file NIHPPrs2579392v3-supplement-1.pdf]

Supplementary Document

Model

The model used here is similar to the SGM developed previously [31–34].

Notation All the vectors and matrices are written in boldface and the scalars are written in normal font. The frequency f of a signal is specified in Hertz (Hz) and the corresponding angular frequency $\omega = 2\pi f$ is used to obtain the Fourier transforms. The connectivity matrix is defined as $\boldsymbol{C} = c_{jk}$, where c_{jk} is the connectivity strength between regions j and k, normalized by the row degree.

Mesoscopic model

For every region k out of the total N regions, we model the local excitatory signal $x_{\mathrm{e}}(t)$, local inhibitory signal $x_{\mathrm{i}}(t)$ as well as the long range excitatory signal $x_k(t)$ where the global connections are incorporated. The local signals $x_{\mathrm{e}}(t)$ and $x_{\mathrm{i}}(t)$ are the same for every region k. They are modeled using an analytical and linearized form of neural mass equations. We write a set of differential equations for evolution of $x_{\mathrm{e}}(t)$ and $x_{\mathrm{i}}(t)$ due to decay of individual signals with a fixed neural gain, incoming signals from coupled excitatory and inhibitory signals, and input white Gaussian noise. Letting $f_e(t)$ and $f_i(t)$ denote the ensemble average neural impulse response functions, the $x_{\mathrm{e}}(t)$ and $x_{\mathrm{i}}(t)$ are modeled as

$$\frac{\mathrm{d}x_{\mathrm{e}}(t)}{\mathrm{d}t} = -\frac{f_{\mathrm{e}}(t)}{\tau_{\mathrm{e}}} \star \big(g_{\mathrm{ee}}\, x_{\mathrm{e}}(t) - g_{\mathrm{ei}}\, f_{\mathrm{i}}(t) \star x_{\mathrm{i}}(t)\big) + p(t)\,, \text{ and,} \tag{2}$$

$$\frac{\mathrm{d}x_{\mathrm{i}}(t)}{\mathrm{d}t} = -\frac{f_{\mathrm{i}}(t)}{\tau_{\mathrm{i}}} \star \big(g_{\mathrm{ii}}\, x_{\mathrm{i}}(t) + g_{\mathrm{ei}}\, f_{\mathrm{e}}(t) \star x_{\mathrm{e}}(t)\big) + p(t)\,, \tag{3}$$

where, $\star$ stands for convolution, $p(t)$ is input noise, parameters g_{ee}, g_{ii}, g_{ei} are neural gain terms, and parameters τ_{e}, τ_{i} are characteristic time constants. These are global parameters and are the same for every region k. Here, the ensemble average neural impulse response functions $f_{\mathrm{e}}(t)$ and $f_{\mathrm{i}}(t)$ are assumed to be Gamma-shaped and written as

$$f_{\mathrm{e}}(t) = \frac{t}{\tau_{\mathrm{e}}^2} \exp\left(\frac{-t}{\tau_{\mathrm{e}}}\right), \quad \text{and,} \quad f_{\mathrm{i}}(t) = \frac{t}{\tau_{\mathrm{i}}^2} \exp\left(\frac{-t}{\tau_{\mathrm{i}}}\right). \tag{4}$$

Macroscopic model

A similar equation is written for the macroscopic signal $x_k(t)$, for every k^{th} region, accounting for long-range excitatory cortico-cortical connections for the pyramidal cells. The evolution of $x_k(t)$ is assumed as a sum of

decay due to individual signals with a fixed excitatory neural gain, incoming signals from all other connected regions determined by the white matter connections, and the input signal $x_\mathrm{e}(t) + x_\mathrm{i}(t)$ determined from Eq. (2) and (3). Signal x_k is modeled as

$$\frac{\mathrm{d}x_k(t)}{\mathrm{d}t} = -\frac{1}{\tau_\mathrm{G}} f_\mathrm{G}(t) \star x_k(t) + \frac{\alpha}{\tau_\mathrm{G}} f_\mathrm{G}(t) \star \sum_{j=1}^{N} c_{jk} x_j(t - \tau_{jk}^v) + \big(x_\mathrm{e}(t) + x_\mathrm{i}(t)\big)\,, \tag{5}$$

where, τ_G is the graph characteristic time constant, α is the global coupling constant, c_{jk} are elements of the connectivity matrix determined from DTI followed by tractography, τ_{jk}^v is the delay in signals reaching from the jth to the kth region, v is the cortico-cortical fiber conduction speed with which the signals are transmitted. The delay τ_{jk}^v is calculated as d_{jk}/v, where d_{jk} is the distance between regions j and k.

This set of equations is parameterized by 8 global parameters: excitatory time constant τ_e, inhibitory time constant τ_i, macroscopic graph time constant τ_G, excitatory neural gain g_ee, inhibitory neural gain g_ii, coupled population neural gain g_ei, global coupling constant α, and conduction speed v. The neural gain g_ee is kept as 1 to ensure parameter identifiability. We estimate the remaining 7 global parameters using an optimization procedure described in the next section.

Model solution in the Fourier domain

Since the above equations are linear, we can obtain a closed-form solution in the Fourier domain as demonstrated below. The Fourier transform $\mathcal{F}()$ is taken at angular frequency ω which is equal to $2\pi f$, where f is the frequency in Hz. The Fourier transform of the equations (2) and (3) will be the following, where $\mathcal{F}(x_\mathrm{e}(t)) = X_\mathrm{e}(\omega)$ and $\mathcal{F}(x_\mathrm{i}(t)) = X_\mathrm{i}(\omega)$, and j is the imaginary unit:

$$\mathrm{j}\omega X_\mathrm{e}(\omega) = -\frac{F_\mathrm{e}(\omega)}{\tau_\mathrm{e}} \left(g_\mathrm{ee} X_\mathrm{e}(\omega) - g_\mathrm{ei} F_\mathrm{i}(\omega) X_\mathrm{i}(\omega)\right) + P(\omega), \text{ and,} \tag{6}$$

$$\mathrm{j}\omega X_\mathrm{i}(\omega) = -\frac{F_\mathrm{i}(\omega)}{\tau_\mathrm{i}} \left(g_\mathrm{ii} X_\mathrm{i}(\omega) + g_\mathrm{ei} F_\mathrm{e}(\omega) X_\mathrm{e}(\omega)\right) + P(\omega)\,. \tag{7}$$

Here, $P(\omega)$ is the Fourier transform of the input Gaussian noise $p(t)$ which we assume to be identically distributed for both the excitatory and inhibitory local populations for each region, and the Fourier transforms of the ensemble average neural response functions are

$$\mathcal{F}(f_\mathrm{e}(t)) = F_\mathrm{e}(\omega) = \frac{1/\tau_\mathrm{e}^2}{(\mathrm{j}\omega + 1/\tau_\mathrm{e})^2}, \quad \text{and,} \quad \mathcal{F}(f_\mathrm{i}(t)) = F_\mathrm{i}(\omega) = \frac{1/\tau_\mathrm{i}^2}{(\mathrm{j}\omega + 1/\tau_\mathrm{i})^2}\,. \tag{8}$$

On solving the above equations (6) and (7), $X_\mathrm{e}(\omega)$ and $X_\mathrm{i}(\omega)$ are

$$X_\mathrm{e}(\omega) = \frac{1 + F_1/(\tau_\mathrm{e} F_2)}{F_3 + F_1^2/(\tau_\mathrm{e}\tau_\mathrm{i} F_2)} P(\omega)\,, \quad \text{and,} \quad X_\mathrm{i}(\omega) = \frac{1 - F_1/(\tau_\mathrm{i} F_3)}{F_2 + F_1^2/(\tau_\mathrm{e}\tau_\mathrm{i} F_3)} P(\omega)\,, \tag{9}$$

where,

$$F_1 = g_\mathrm{ei} F_\mathrm{e}(\omega) F_\mathrm{i}(\omega)\,, \quad F_2 = \mathrm{j}\omega + \frac{g_\mathrm{ii}}{\tau_\mathrm{i}} F_\mathrm{i}(\omega)\,, \quad \text{and,} \quad F_3 = \mathrm{j}\omega + \frac{g_\mathrm{ee}}{\tau_\mathrm{e}} F_\mathrm{e}(\omega)\,. \tag{10}$$

Then, the transfer functions $H_\mathrm{e}(\omega)$ and $H_\mathrm{i}(\omega)$ can be separated out and we get

$$X_\mathrm{e}(\omega) = H_\mathrm{e}(\omega) P(\omega)\,, \quad \text{and} \quad X_\mathrm{i}(\omega) = H_\mathrm{i}(\omega) P(\omega)\,. \tag{11}$$

The total neural population is therefore

$$X_{\text{local}}(\omega) = (H_{\text{e}}(\omega) + H_{\text{i}}(\omega))P(\omega)\,, \tag{12}$$

thus, $H_{\text{local}}(\omega) = H_{\text{e}}(\omega) + H_{\text{i}}(\omega)$.

In order to obtain a Fourier response of the macroscopic signal, we first re-write Eq. (5) in the vector form

$$\frac{\mathrm{d}\boldsymbol{x}(t)}{\mathrm{d}t} = -\frac{1}{\tau_{\text{G}}} f_{\text{G}}(t) \star \boldsymbol{x}(t) + \frac{\alpha}{\tau_{\text{G}}} f_{\text{G}}(t) \star \boldsymbol{C}\boldsymbol{x}(t - \tau^{v}_{jk}) + \boldsymbol{x}_{\text{local}}(t)\,. \tag{13}$$

We similarly take a Fourier response of the macroscopic signal and obtain the following as the Fourier transform of Eq. (13), where $\mathcal{F}(\boldsymbol{x}(t)) = \boldsymbol{X}(\omega)$:

$$\mathrm{j}\omega \boldsymbol{X}(\omega) = -\frac{1}{\tau_{\text{G}}} F_{\text{G}}(\omega)\boldsymbol{X}(\omega) + \frac{\alpha}{\tau_{\text{G}}} F_{\text{G}}(\omega)\boldsymbol{C}^{*}(\omega)\boldsymbol{X}(\omega) + \boldsymbol{X}_{\text{local}}(\omega)\,, \tag{14}$$

where, $\boldsymbol{C}^{*}(\omega) \equiv [c_{ij} \exp\left(-j\omega\tau^{v}_{ij}\right)]$. Note that each element in the matrix $\boldsymbol{C}$ is normalized already by the row degree. The above equation can be re-arranged to give

$$\left(\mathrm{j}\omega + \frac{1}{\tau_{\text{G}}} F_{\text{G}}(\omega)(\boldsymbol{I} - \alpha \boldsymbol{C}^{*}(\omega))\right) \boldsymbol{X}(\omega) = H_{\text{local}}(\omega)\boldsymbol{P}(\omega)\,. \tag{15}$$

Here, we define the complex Laplacian matrix:

$$\boldsymbol{\mathcal{L}}(\omega) = \boldsymbol{I} - \alpha \boldsymbol{C}^{*}(\omega)\,, \tag{16}$$

where, I is the identity matrix of size $N \times N$. The eigen-decomposition of this complex Laplacian matrix is

$$\boldsymbol{\mathcal{L}}(\omega) = \boldsymbol{U}(\omega)\boldsymbol{\Lambda}(\omega)\boldsymbol{U}(\omega)^{\text{H}}\,, \tag{17}$$

where, $\boldsymbol{U}(\omega)$ are the eigenmodes/eigenvectors and $\boldsymbol{\Lambda}(\omega) = \text{diag}([\lambda_1(\omega), \ldots, \lambda_N(\omega)])$ consist of the eigenvalues $\lambda_1(\omega), \ldots, \lambda_N(\omega)$, at angular frequency ω. The macroscopic response $\boldsymbol{X}(\omega)$ from Eq. (15) becomes

$$\boldsymbol{X}(\omega) = \left(\mathrm{j}\omega + \frac{1}{\tau_{\text{G}}} F_{\text{G}}(\omega)\boldsymbol{\mathcal{L}}(\omega)\right)^{-1} H_{\text{local}}(\omega)\boldsymbol{P}(\omega)\,. \tag{18}$$

By using the eigen-decomposition of the Laplacian matrix, this yields

$$\boldsymbol{X}(\omega) = \sum_{k=1}^{N} \frac{\boldsymbol{u}_k(\omega)\boldsymbol{u}_k(\omega)^{\text{H}}}{\mathrm{j}\omega + \tau_{\text{G}}^{-1}\lambda_k(\omega)F_{\text{G}}(\omega)} H_{\text{local}}(\omega)\boldsymbol{P}(\omega)\,, \tag{19}$$

where, $\boldsymbol{u}_k(\omega)$ are the eigenmodes from $\boldsymbol{U}(\omega)$ and $\lambda_k(\omega)$ are the eigenvalues from $\boldsymbol{\Lambda}(\omega)$ obtained by the eigen-decomposition of the complex Laplacian matrix $\boldsymbol{\mathcal{L}}(\omega)$ obtained in Eq. 17. Equation (19) is the closed-form steady state solution of the macroscopic signals at a specific angular frequency ω. We use this modeled spectra to compare against empirical MEG spectra and subsequently estimate model parameters. Overall workflow is shown in Fig. S1. The structural connectome and the distance matrix are shown in Fig. S5 and S6, respectively.

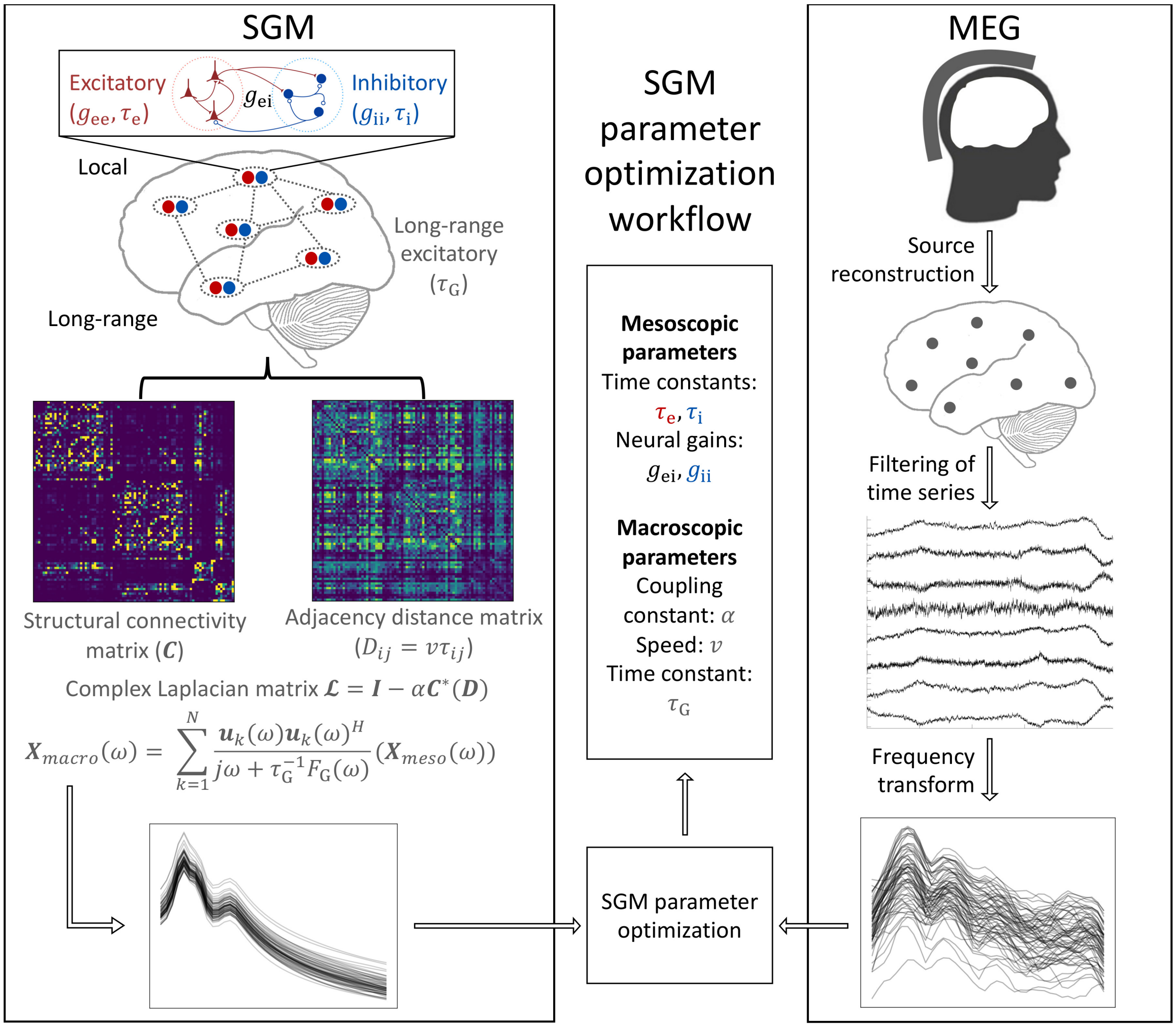

Figure S1: SGM models excitatory and inhibitory neuronal subpopulation signals that influence the long-range excitatory signals. The long-range signals are connected to each other via the structural connectome, and these signals transmit with a fixed conduction speed. SGM provides a closed-form solution in the frequency domain. This is compared to the frequency spectra obtained from MEG for inferring the SGM model parameters.

Model performance

Fig. S2 shows the regional and band-specific correlations. Fig. S2A shows the regional spectral correlations for healthy controls (top) and patients with AD (bottom). The spectral correlations are greater than 0.5 for most subjects and regions. Precisely, the spectral correlations were greater than 0.5 for more than 90% and 93% of controls and AD, respectively. Fig. S2B shows the spatial correlations for the delta-theta, alpha, and beta frequency bands for healthy controls (left) and patients with AD (right). The spatial correlations are also greater than 0.5 for most subjects and frequency bands. Precisely, the spatial correlations were greater than 0.5 for more than 94% and 97% of controls and AD, respectively.

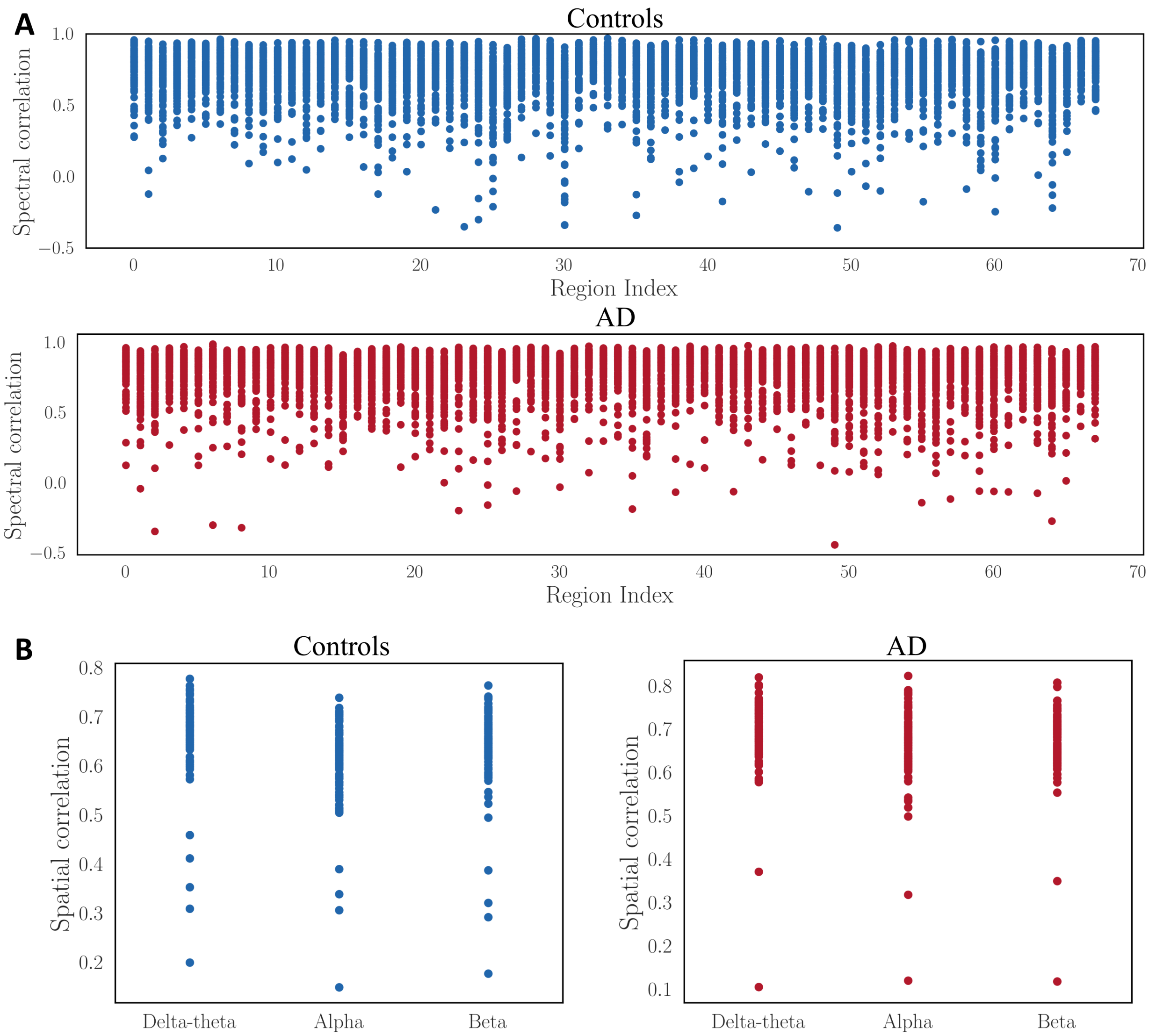

Figure S2: **A:** Regional spatial correlations for healthy controls (top) and patients with AD (bottom). Every point indicates the spectral correlation for a specific subject in a brain region indexed in the x-axis. **B:** Spatial correlations for healthy controls (left) and patients with AD (right). Every point indicates the spatial correlation for a specific subject in a frequency band indexed in the x-axis.

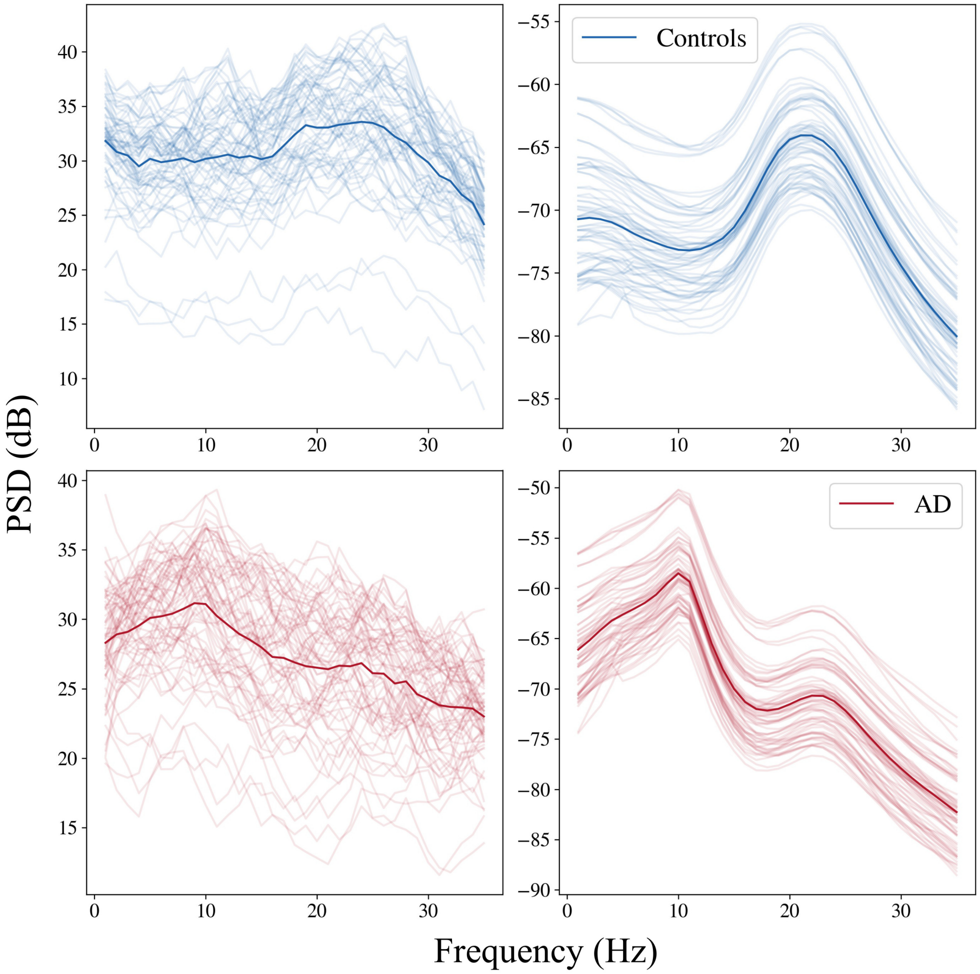

Figure S3: Empirical (left) and SGM (right) PSD for control (top) and AD (bottom) for specific subjects with spectral correlation approximately equal to the mean correlation. The spectral correlation for control was 0.72 and for AD was 0.78 for the data shown here.

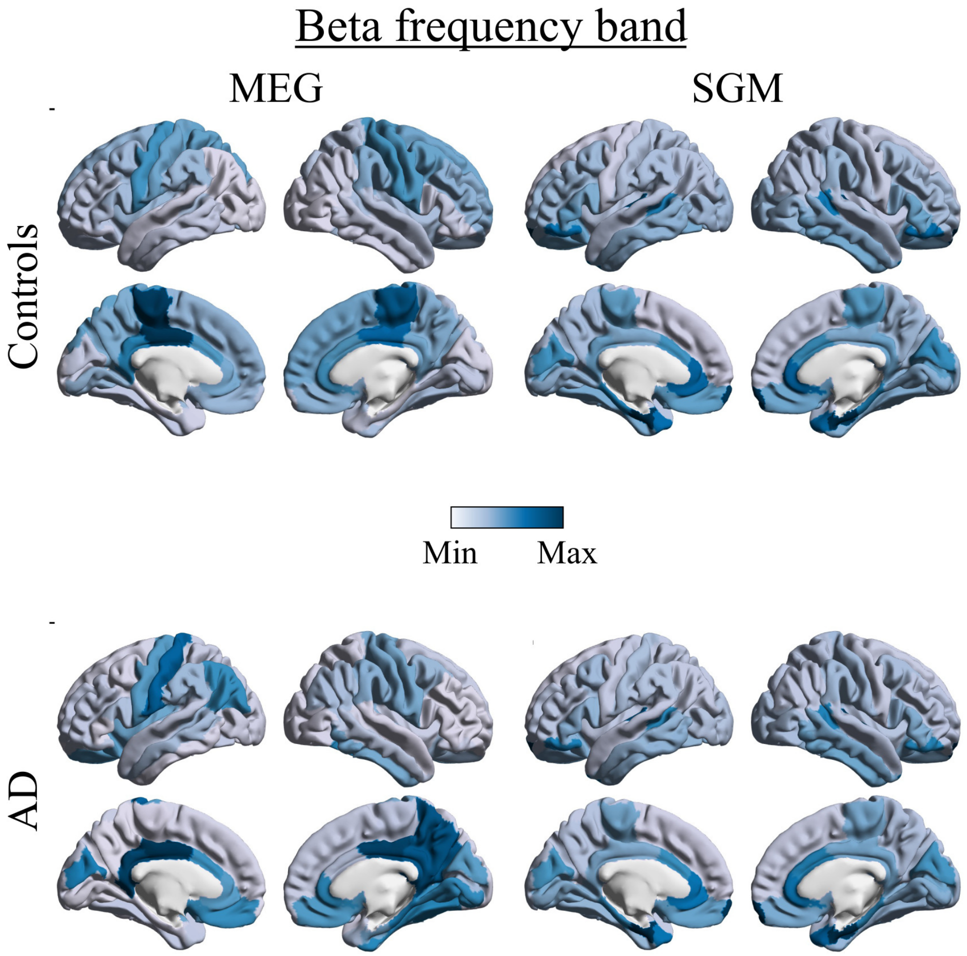

Figure S4: Spatial pattern of the spectral power in the delta-theta frequency band for subjects with spatial correlation in the delta-theta band approximately equal to the mean correlation. Top for controls and bottom for AD. Left for MEG and right for SGM. The minimum and maximum values were based on the dynamic range for each of them. The spatial correlation for control was 0.63 and for AD was 0.67 for the data shown here.

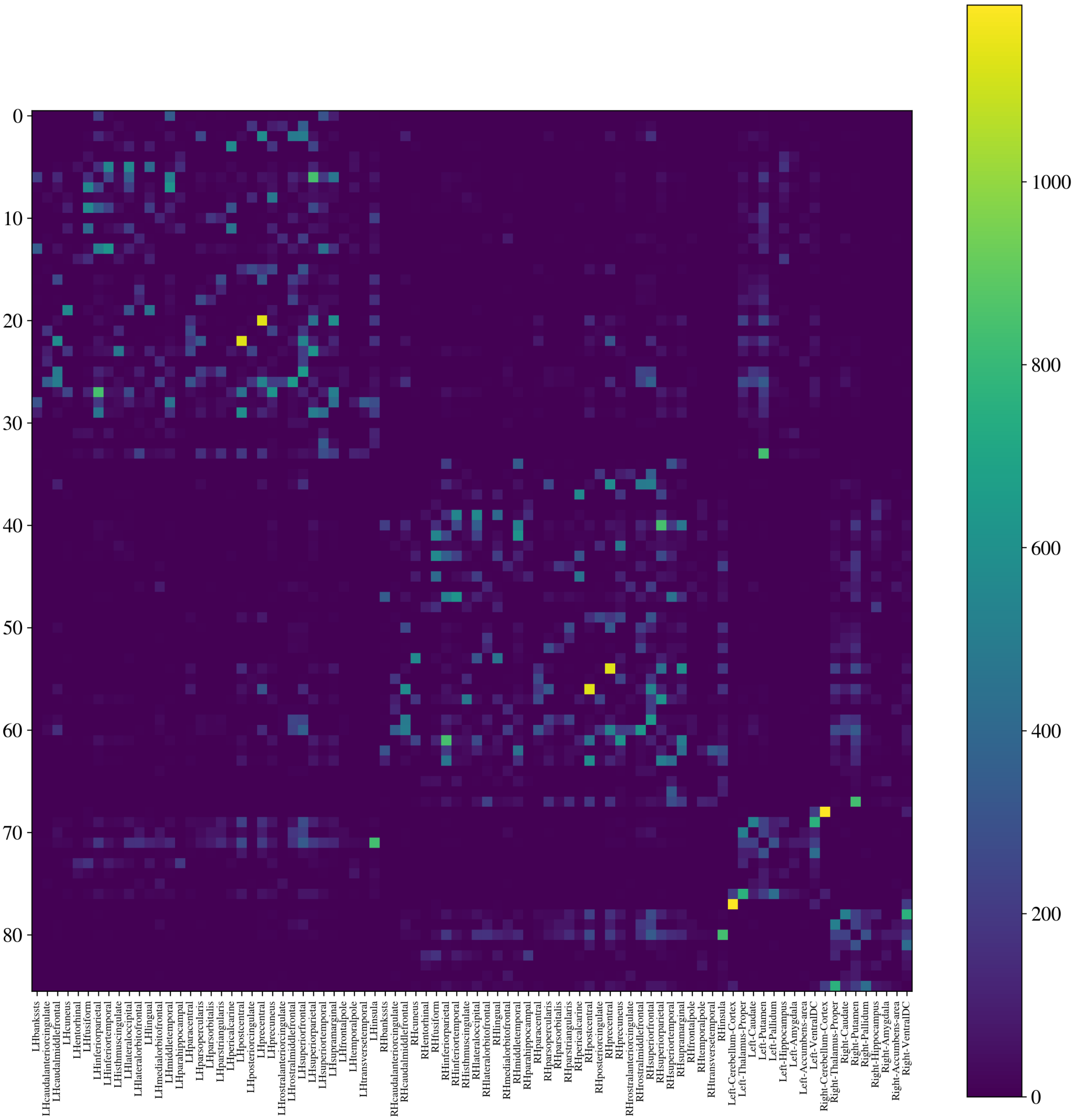

Figure S5: Structural connectivity matrix

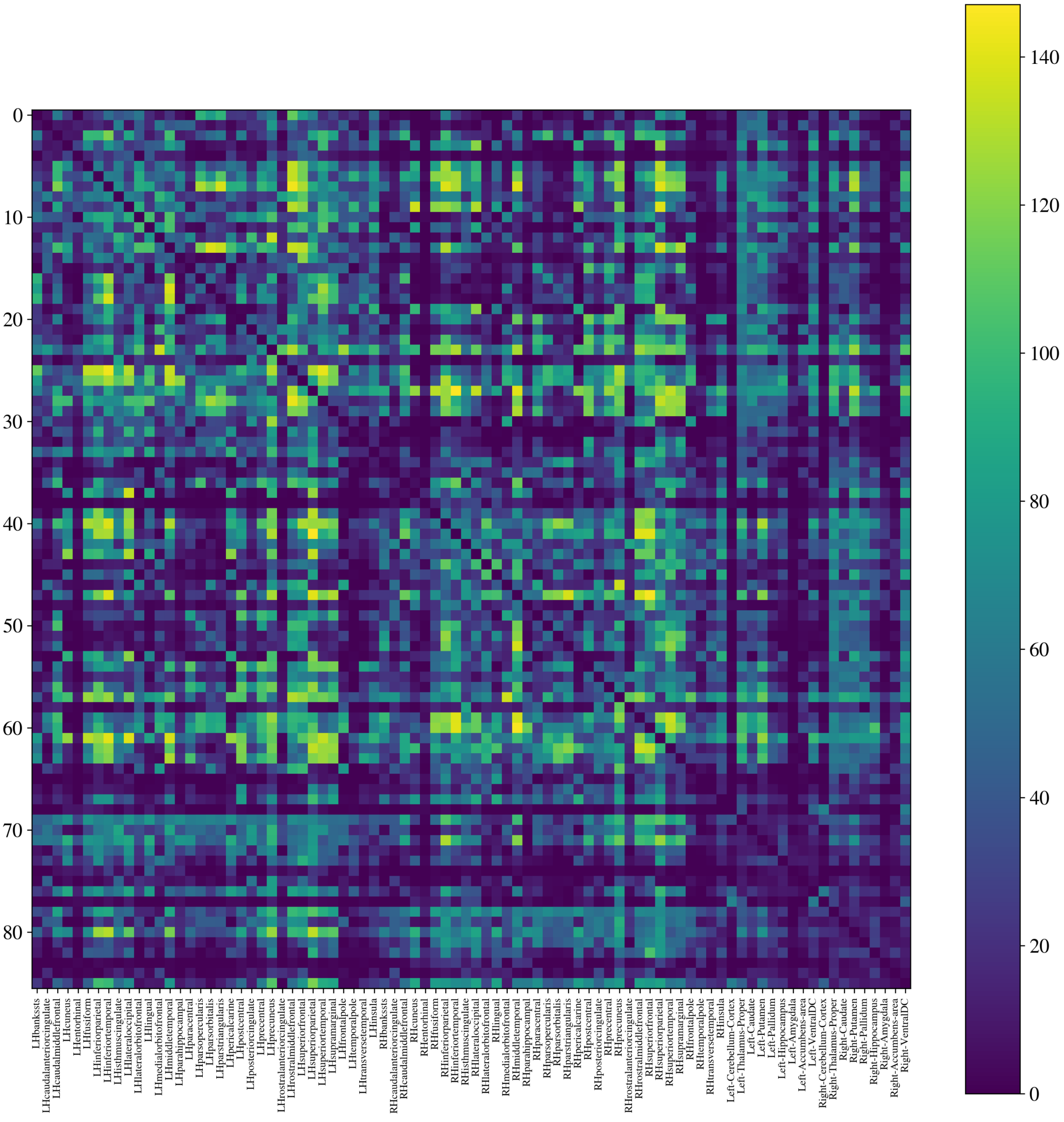

Figure S6: Distance matrix

Table S1: Linear Regression Results for MMSE vs tauG

**Dep. Variable:**	MMSE	**R-squared:**	0.111
**Model:**	OLS	**Adj. R-squared:**	0.101
**Method:**	Least Squares	**F-statistic:**	10.63
**No. Observations:**	87	**Prob (F-statistic):**	0.00160
**Df Residuals:**	85	**Log-Likelihood:**	-267.43
**Df Model:**	1	**AIC:**	538.9
**Covariance Type:**	nonrobust	**BIC:**	543.8

	**coef**	**std err**	**t**	**P> |t|**	**[0.025**	**0.975]**
**const**	25.7678	1.250	20.619	0.000	23.283	28.253
**tauG**	-0.2582	0.079	-3.260	0.002	-0.416	-0.101

**Omnibus:**	24.622	**Durbin-Watson:**	1.512
**Prob(Omnibus):**	0.000	**Jarque-Bera (JB):**	36.960
**Skew:**	-1.221	**Prob(JB):**	9.42e-09
**Kurtosis:**	5.057	**Cond. No.**	34.9

Table S2: Multivariate Linear Regression Results

**Dep. Variable:**	MMSE	**R-squared:**	0.162
**Model:**	OLS	**Adj. R-squared:**	0.121
**Method:**	Least Squares	**F-statistic:**	3.961
**No. Observations:**	87	**Prob (F-statistic):**	0.00546
**Df Residuals:**	82	**Log-Likelihood:**	-264.87
**Df Model:**	4	**AIC:**	539.7
**Covariance Type:**	nonrobust	**BIC:**	552.1

	**coef**	**std err**	**t**	**P> |t|**	**[0.025**	**0.975]**
**const**	20.9932	4.557	4.606	0.000	11.927	30.059
**age**	0.0734	0.066	1.107	0.272	-0.059	0.205
**tauG**	-0.2243	0.082	-2.745	0.007	-0.387	-0.062
**taue**	-0.0947	0.068	-1.394	0.167	-0.230	0.040
**gii**	2.3633	1.228	1.925	0.058	-0.079	4.805

**Omnibus:**	26.612	**Durbin-Watson:**	1.578
**Prob(Omnibus):**	0.000	**Jarque-Bera (JB):**	42.646
**Skew:**	-1.274	**Prob(JB):**	5.49e-10
**Kurtosis:**	5.297	**Cond. No.**	541.

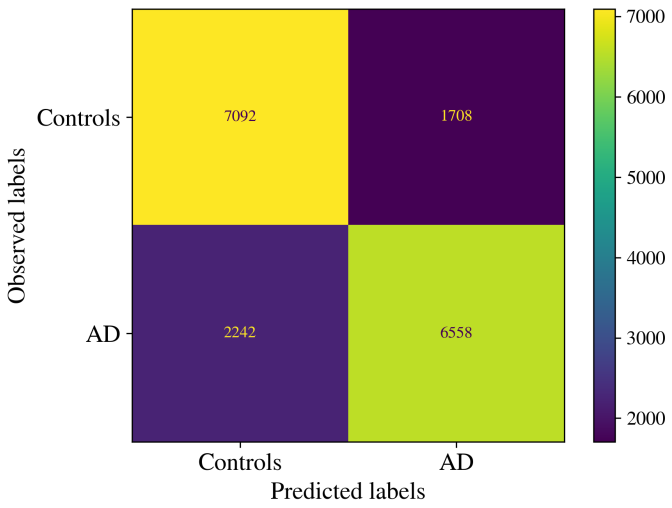

Figure S7: Confusion matrix from classification of AD versus controls
